# Patient stratification using plasma cytokines and their regulators in sepsis: relationship to outcomes, treatment effect and leucocyte transcriptomic subphenotypes

**DOI:** 10.1136/thorax-2023-220538

**Published:** 2024-03-12

**Authors:** David Benjamin Antcliffe, Yuxin Mi, Shalini Santhakumaran, Katie L Burnham, A Toby Prevost, Josie K Ward, Timothy J Marshall, Claire Bradley, Farah Al-Beidh, Paula Hutton, Stuart McKechnie, Emma E Davenport, Charles J Hinds, Cecilia M O'Kane, Daniel Francis McAuley, Manu Shankar-Hari, Anthony C Gordon, Julian C Knight

**Affiliations:** 1 Division of Anaesthetics, Pain Medicine and Intensive Care, Department of Surgery and Cancer, Faculty of Medicine, Imperial College London, London, UK; 2 Centre for Perioperative and Critical Care Research, Imperial College Healthcare NHS Trust, London, UK; 3 Wellcome Centre for Human Genetics, Nuffield Department of Medicine, University of Oxford, Oxford, UK; 4 Imperial Clinical Trials Unit, School of Public Health, Imperial College London, London, UK; 5 Wellcome Sanger Institute, Wellcome Genome Campus, Hinxton, UK; 6 Nightingale-Saunders Clinical Trials and Epidemiology Unit, King's College London, London, UK; 7 Conway Institute, School of Medicine, University College Dublin, Dublin, Ireland; 8 Department of Anaesthetics, Royal Prince Alfred Hospital, Camperdown, New South Wales, Australia; 9 Central Clinical School Faculty of Medicine and Health, The University of Sydney, Sydney, New South Wales, Australia; 10 Adult Intensive Care Unit, John Radcliffe Hospital, Oxford, UK; 11 William Harvey Research Institute, Barts & The London School of Medicine and Dentistry, Queen Mary University of London, London, UK; 12 Wellcome-Wolfson Institute for Experimental Medicine, Queen's University Belfast, Belfast, UK; 13 Regional Intensive Care Unit, Royal Victoria Hospital, Belfast, UK; 14 Northern Ireland Clinical Trials Unit, Royal Hospitals, Belfast, UK; 15 The Queen’s Medical Research Institute, The University of Edinburgh College of Medicine and Veterinary Medicine, Edinburgh, UK; 16 Intensive Care Unit, Royal Infirmary of Edinburgh, Edinburgh, UK; 17 Chinese Academy of Medical Science Oxford Institute, University of Oxford, Oxford, UK

**Keywords:** respiratory infection, bacterial infection, critical care

## Abstract

**Rationale:**

Heterogeneity of the host response within sepsis, acute respiratory distress syndrome (ARDS) and more widely critical illness, limits discovery and targeting of immunomodulatory therapies. Clustering approaches using clinical and circulating biomarkers have defined hyper-inflammatory and hypo-inflammatory subphenotypes in ARDS associated with differential treatment response. It is unknown if similar subphenotypes exist in sepsis populations where leucocyte transcriptomic-defined subphenotypes have been reported.

**Objectives:**

We investigated whether inflammatory clusters based on cytokine protein abundance were seen in sepsis, and the relationships with previously described transcriptomic subphenotypes.

**Methods:**

Hierarchical cluster and latent class analysis were applied to an observational study (UK Genomic Advances in Sepsis (GAinS)) (n=124 patients) and two clinical trial datasets (VANISH, n=155 and LeoPARDS, n=484) in which the plasma protein abundance of 65, 21, 11 circulating cytokines, cytokine receptors and regulators were quantified. Clinical features, outcomes, response to trial treatments and assignment to transcriptomic subphenotypes were compared between inflammatory clusters.

**Measurements and main results:**

We identified two (UK GAinS, VANISH) or three (LeoPARDS) inflammatory clusters. A group with high levels of pro-inflammatory and anti-inflammatory cytokines was seen that was associated with worse organ dysfunction and survival. No interaction between inflammatory clusters and trial treatment response was found. We found variable overlap of inflammatory clusters and leucocyte transcriptomic subphenotypes.

**Conclusions:**

These findings demonstrate that differences in response at the level of cytokine biology show clustering related to severity, but not treatment response, and may provide complementary information to transcriptomic sepsis subphenotypes.

**Trial registration number:**

ISRCTN20769191, ISRCTN12776039.

**WHAT IS ALREADY KNOWN ON THIS TOPIC**

- Acute respiratory distress syndrome has been described by two inflammatory subphenotypes that are associated with disease severity, outcome and treatment effect.
- Similarly, a number of sepsis leucocyte transcriptomic subphenotypes have been described with similar associations; however, little is not known about how inflammatory subphenotypes in sepsis relate to transcriptomic subphenotypes.

**WHAT THIS STUDY ADDS**

- We demonstrate that across three independent datasets a group with high levels of pro-inflammatory and anti-inflammatory cytokines exists that is associated with worse organ dysfunction and survival.
- We found variable overlap of inflammatory clusters and leucocyte transcriptomic subphenotypes demonstrating the complementary nature of these two approaches.

**HOW THIS STUDY MIGHT AFFECT RESEARCH, PRACTICE OR POLICY**

- These results demonstrate that plasma cytokine-derived clusters have utility for stratifying patients with sepsis but that an optimal approach to define sepsis subphenotypes may need to account for both cytokine protein abundance and transcriptomic data.

## Introduction

Sepsis is defined as life-threatening organ dysfunction caused by a dysregulated host response to infection^1^ and causes 11 million deaths worldwide annually.^2^ Sepsis is a heterogeneous syndrome, which is a barrier to finding novel therapies,^3 4^ prompting a search for subpopulations (also known as subphenotypes), with the hope that patients could be stratified for targeted treatments. Such heterogeneity is seen across critical illness syndromes. The identification of potentially treatable traits underlying observed syndromes offers the opportunity to revisit current disease classifications.^5^

In sepsis, several approaches have been used to identify subphenotypes, including using clinical,^6 7^ gene expression^8–10^ and cytokine^11–16^ data. In acute respiratory distress syndrome (ARDS), latent class analysis using clinical and plasma biomarker data including cytokines has consistently identified two subphenotypes. A hyper-inflammatory subphenotype is characterised by higher cytokine levels, shock, worse clinical outcomes and potentially different responses to treatment compared with the hypo-inflammatory subphenotype.^17–21^ The extent to which such a phenomenon is also seen in sepsis is less clear. We have previously demonstrated how differences in the white blood cell transcriptome allow patient stratification based on sepsis response signatures (SRS)^8 9 22 23^ but how they relate with plasma cytokine-derived clusters is unknown.

Here, we investigate inflammatory subgroups in sepsis and explore the relationships of these with heterogeneity of treatment effect and previously described transcriptomic subphenotypes.

A version of this manuscript has been made available on a preprint server^24^ (https://doi.org/10.1101/2022.07.12.22277463).

## Methods

### Patients

Blood samples and clinical data were available from the VANISH^25^ and LeoPARDS^26^ septic shock trials, which included patients with septic shock from any source, and the UK Genomic Advances in Sepsis (GAinS)^8 27^ study, which included patients with sepsis due to community-acquired pneumonia (CAP) or faecal peritonitis (FP).

VANISH was a factorial (2×2) randomised trial comparing vasopressin with norepinephrine and hydrocortisone with placebo in septic shock that recruited patients between 2013 and 2015. The LeoPARDS trial was a randomised trial comparing levosimendan with placebo in septic shock that recruited patients between 2014 and 2015. In LeoPARDS and VANISH, all patients were included who had appropriate baseline blood samples. GAinS was an observational study of patients admitted to intensive care unit (ICU) with sepsis or septic shock. From this cohort, samples were selected for plasma protein measurement considering SRS assignment availability and demographics matching those of the original cohort in which SRS was defined. Ninety-six patients with CAP and 32 patients with FP recruited between 2006 and 2015 were included. Descriptions of these studies can be found in the online supplemental file.

10.1136/thorax-2023-220538.supp1 Supplementary data

### Measurement of plasma protein cytokines, cytokine receptors and regulators

Plasma was collected on the first day of ICU admission (GAinS) or of septic shock (VANISH and LeoPARDS). Samples were separated locally, frozen according to standardised operating procedures and sent to the co-ordinating centres for storage at −80°C and analysis. Assays were conducted blinded to treatment allocation and outcome.

Overall, 70 plasma proteins involved in cytokine inflammatory biology were assayed, including 65 cytokines (of which 19 were chemokines), 2 cytokine receptors, 2 enzyme regulators (matrix metalloproteinase (MMP)1), myeloperoxidase (MPO)) and the inflammatory regulator glycoprotein intercellular adhesion molecule (ICAM). These are referred to here on as plasma cytokines and related proteins or abbreviated as ‘plasma cytokines’, with 65 of the proteins measured in GAinS, 21 in VANISH and 11 in LeoPARDS. Details of the specific proteins and assays are in the online supplemental file.

Protein measurements were regarded as missing if no measurement could be obtained due to technical failures. These occurred at random and were not imputed.

### Hierarchical cluster analysis

**Figure 1:**
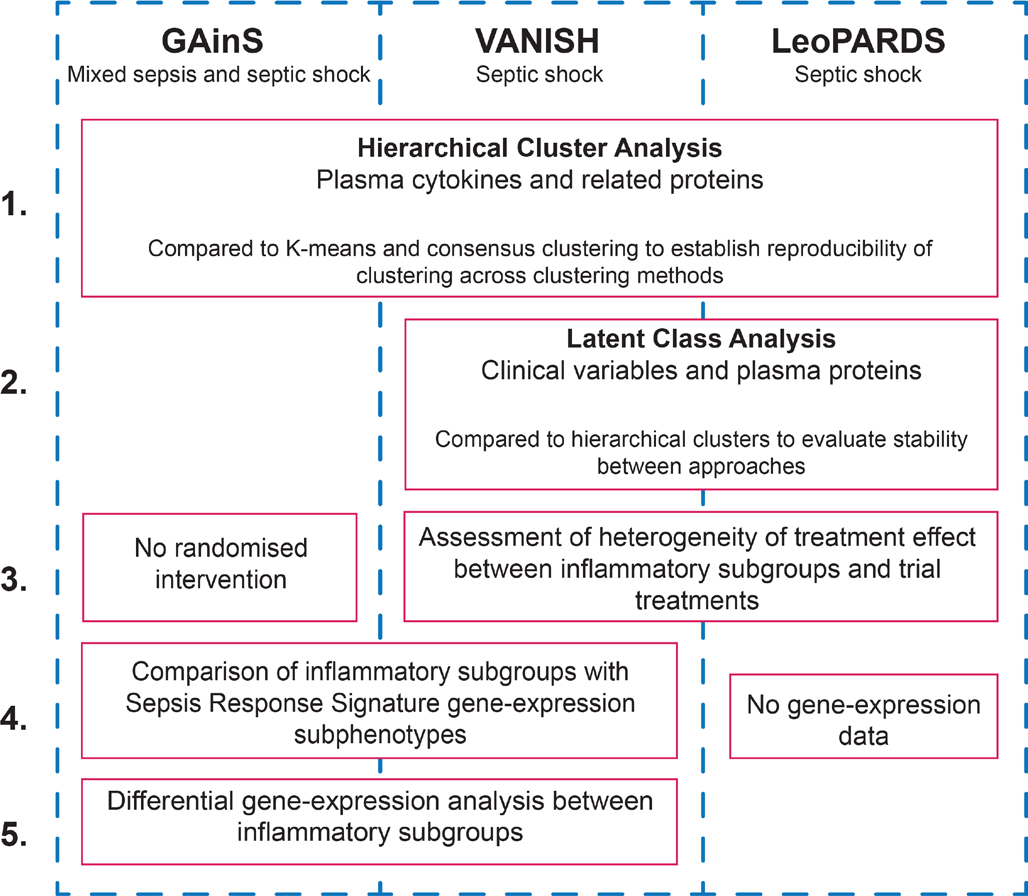
Diagram demonstrating the analyses that were performed across the three datasets and the comparisons that were made. Each column represents a dataset with the red boxes demonstrating the analysis that was performed, with the columns they span reflecting the cohorts the analysis was performed on. GAinS, Genomic Advances in Sepsis.

Full details can be found in the online supplemental file. Briefly, hierarchical cluster analysis was applied to the plasma cytokine panels used in GAinS, VANISH and LeoPARDS independently (figure 1). As hierarchical cluster analysis is unable to handle missing data and variables missing due to technical issues occurred at random, only patients with complete panels for a given cohort were included. Where measurements were obtained but were outside the limits of quantification, these were censored at the quantification limit based on the standard curve for the analysis run. Plasma protein concentrations were natural log transformed prior to clustering. Dissimilarity between samples was measured by Euclidean distance. Ward’s method was used as linkage for cluster agglomeration using the hclust function. We also deployed k-means clustering and consensus clustering^28^ to help determine the optimal number of clusters and assess reproducibility. Optimum number of clusters was determined using several methods including inspection of the dendrograms, the cumulative distribution of the consensus index in each cluster number and using the NbClust package.^29^ Clustering was performed in R^30^ and the final cluster assignments were derived using the hclust function as these are highly concordant with consensus clusters but with a more accessible and repeatable algorithm and clear interpretation.

### Latent class analysis

Latent class analysis has previously been caried out independently on the LeoPARDS and VANISH datasets (figure 1) and is described in full in a report to the funding body that has supported some of the work^31^ and is described in detail in the online supplemental methods. These datasets were used for latent class analysis as both had the same subset of plasma cytokines and related proteins assayed and clinical variables recorded, and being interventional trials, allowed exploration of subphenotype and treatment interaction. Variables were chosen for inclusion based on their associations with sepsis pathophysiology (PaO_2_/FiO_2_ ratio, creatinine, platelets, bilirubin, lactate, interleukin (IL)-1β, IL-6, IL-8, IL-10, IL-17, IL-18, MPO, soluble ICAM, ANG-2, sTNFr, MCP-1 (CCL2), troponin and NT-proBNP). Plasma protein abundances were log transformed and standardised. The Bayesian Information Criterion, the Akaike Information Criterion, log likelihood, entropy,^32^ class sizes and the mean probability of class assignment, averaged over participants in the class were used to assess the optimal number of classes. Models were fit using the gsem package in STATA V.15 (StataCorp, College Station, Texas, USA).

### Clinical outcomes and assessment of heterogeneity of treatment effect

The primary outcome on which to explore heterogeneity of treatment effect, for the data from the LeoPARDS trial, was survival at 3 months. Mean total SOFA score over 28 days (or ICU stay, whichever was shorter) and survival to 28 days were examined as secondary outcomes. For the VANISH trial, we examined survival to 28 days, 3-month survival was not available. Survival free of renal failure to 28 days among patients not in renal failure at baseline, and days alive and free of renal failure up to 28 days for all other patients (those who died or experienced some renal failure by day 28) were also examined.

Treatment effect was assessed primarily on an intention to treat basis, with the exception of hydrocortisone versus placebo, in the VANISH trial as patients were only eligible to receive hydrocortisone/placebo if they reached the maximum infusion of the first study drug so only patients eligible to receive the second drug were included for this comparison.

Heterogeneity of treatment effect was assessed between hierarchical cluster analysis clusters using binary logistic regression for 28-day and 90-day outcomes, the aligned rank transform test^33 34^ for renal failure free days as the assumptions of linear regression were not met and logistic regression for square root transformed mean total SOFA, as described previously.^22^ In all analyses, an interaction term was included between cluster and drug allocation with a p value of <0.05 for the interaction term representing a differential treatment effect. Assessment of heterogeneity of treatment effect based on latent classes has been described previously.^31^

### Transcriptomic subphenotypes and differential gene expression

Genome-wide blood leucocyte transcriptomic data, using Illumina Human-HT-12 v4 Expression BeadChips (47 323 probes), were available from the VANISH^35^ and GAinS^36–39^ studies as described previously.^8 9 22^ Blood samples for transcriptomic analysis were collected using LeukoLOCK filters (GAinS) and PAXgene tubes (VANISH) at the same time that plasma was collected. SRS subphenotypes have previously been assigned as described previously.^8 9 22^

After co-normalisation, batch correction and additional quality control, there were 28 220 communal probes in 115 patients in GAinS and 149 in VANISH from those included in the hierarchical cluster analysis of plasma cytokine and related proteins. Differentially expressed genes between subphenotypes were identified using the limma package^40^ as those with both a false discovery rate (FDR) <0.05 and fold change >1.5 in accordance with previous work.^8 9^ Pathway enrichment analysis was performed with the R package XGR,^41^ using annotations of Gene Ontology Biological Process. Contrasts between cytokine clusters or SRS groups were limited to the same subsets of patients with both assignments available. More detail can be found in the online supplemental file.

### Other statistical analysis

Data were compared using the Mann-Whitney U test, Kruskal-Wallis test, χ^2^ test or Fisher’s exact test (Fisher’s exact test was used when the number of events was <10) as appropriate. The Benjamini-Hochberg procedure was applied for comparisons of plasma cytokines and related proteins between hierarchical cluster analysis derived and SRS groups, but as this was an exploratory analysis p values for clinical comparisons were not corrected. All tests were two-sided and a p value or FDR <0.05 was taken as statistical significance. Correlation was evaluated with Pearson’s or Spearman’s rank correlation coefficients. For principal component analysis, variables were natural log transformed and then zero-centred, without further scaling. Where data allowed, patients were also assigned to inflammatory groups using the previously published parsimonious ARDS models^42^ and these allocations were compared with latent class and hierarchical cluster analysis groups. Statistical analysis was performed in R,^30^ SPSS V.25 (IBM, USA) and STATA V.17 (StataCorp).

## Results

The abundance of plasma cytokine and related proteins (cytokine receptors, enzyme and inflammatory regulators) was available for 176/409 patients from VANISH and 493/516 from LeoPARDS. Included patients were similar to those not contributing samples (online supplemental table S1). In GAinS, 4 CAP samples failed quality control and were excluded from clustering analysis, leaving 124 patients. Details of the circulating plasma cytokines and related proteins assayed, and the proportions below the level of quantification are provided in online supplemental table S2.

### Hierarchical clustering analysis of plasma cytokine and related proteins reveals up to three patient clusters in sepsis

**Figure 2:**
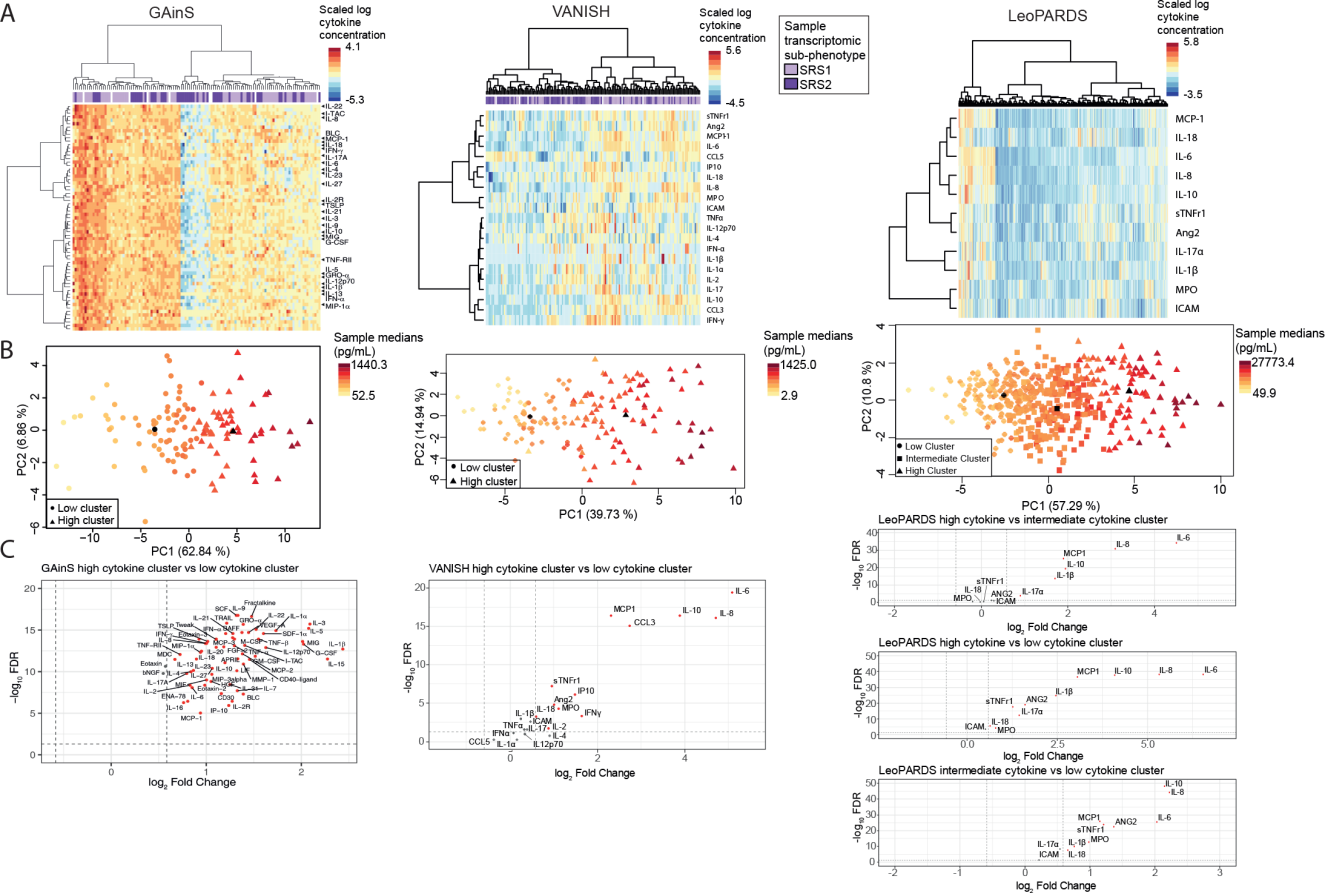
Unsupervised patient structure in Genomic Advances in Sepsis (GAinS) (n=124, left), VANISH (n=155, middle) and LeoPARDS (n=484, right). (A) Heatmaps and cluster dendrograms from hierarchical clustering, patients shown as columns and plasma cytokines and related proteins as rows. Clustering was performed on natural logarithm transformed plasma protein concentrations but for clarity of presentation heatmaps are displayed as z-scaled values. Solid bars represent sepsis response signature (SRS) assignments in GAinS and VANISH (light purple=SRS1, dark purple=SRS2, blank=no assignment available). A full-size version of the heatmap in GAinS where all plasma proteins are labelled and heatmaps from all datasets where non-scaled concentrations are plotted are available as online supplemental figure S2. (B) Principal component analysis scores plot where data points are shaded based on median concentration of all plasma cytokines and related proteins measured in each sample, and the symbol shapes represent the cluster assignments (circles=low cytokine cluster, squares=intermediate cytokine cluster, triangles=high cytokine cluster, centroids for each group are shown with the relevant symbol in black). Percentage of variance explained by the principal components (PC) 1/2 are stated in parentheses. (C) Volcano plots comparing high cytokine clusters with low cytokine clusters (GAinS and VANISH), where the false discovery rate (FDR) is obtained from a Mann-Whitney U test with a Benjamini-Hochberg correction, or high versus low, high versus intermediate and intermediate versus low clusters (LeoPARDS), where FDR is obtained from a post hoc, pairwise Wilcoxon test with Benjamini-Hochberg correction for both the number of pairwise comparisons and the number of plasma proteins compared.

Overall, hierarchical cluster analysis best described VANISH and GAinS by two classes (figure 2, online supplemental figures S1 and S2) with consensus clustering based on hierarchical cluster analysis producing highly consistent assignments (94% for VANISH, 100% for GAinS, online supplemental figure S1). In GAinS, a two-cluster model was supported by seven metrics from NbClust, more than any other number of clusters. In VANISH, two or three cluster structures were suggested by eight metrics each. As the dataset was small and a third cluster would only contain 27 members, we retained the two-cluster model. LeoPARDS, the largest dataset, was described by either two or three clusters (figure 2, online supplemental figures S1 and S2) with the most consistent cluster assignment between hierarchical cluster analysis and consensus clustering being with a three-class model (91% vs 86%), this was also supported by NbClust where three clusters were suggested by 10 metrics.

Principal component analysis showed separation of the classes along the first component in the three cohorts (figure 2), which was strongly correlated with the median concentration of the plasma cytokine and related proteins measured in each sample (Spearman’s rho=0.982/0.904/0.896 for GAinS/VANISH/LeoPARDS). In all cohorts, we observed a ‘high cytokine’ cluster that had higher concentrations of almost all assayed plasma proteins than a ‘low cytokine’ cluster (online supplemental table S3, figure 2), including those with anti-inflammatory functions such as IL-10, IL-2R, TNF-RII and sTNFr1. The third cluster in LeoPARDS was intermediate between the others.

Patients in the high cytokine clusters showed features of more severe disease than patients in the other clusters, such as higher heart rates, lower PaO_2_:FiO_2_ ratios and higher requirements for fluid and norepinephrine (online supplemental table S4). Mortality at 28 days was highest in the high cytokine groups in VANISH (36% vs 20%, p=0.03) and LeoPARDS (LHC 45% vs LIC 34% vs LLC 24%, p=0.002) with the same trend in GAinS (online supplemental table S4). Cluster membership only showed association with source of infection in LeoPARDS, where the low cytokine group had the most cases of pneumonia and least of intra-abdominal infection (online supplemental table S4).

### Evidence for concordant patient classes from latent class analysis supports a stratified sepsis state based on plasma cytokines and related proteins

To explore the robustness of subphenotypes derived from hierarchical cluster analysis of plasma cytokine and related proteins, these were compared with those derived from latent class analysis of a combination of inflammatory and clinical variables, an approach which accommodates different data types, handles missing data and has been applied extensively in ARDS.^17–21^ We only applied latent class analysis in the LeoPARDS and VANISH cohorts since not all variables required for the latent class analysis model were available in GAinS, and that one primary aim of the latent class analysis was to analyse the heterogeneous treatment effect in interventional trials. As latent class analysis is able to handle missing data, it also allowed more patients to be included in the analysis than hierarchical cluster analysis (493 patients in LeoPARDS and 176 in VANISH). These latent class analysis results have been previously detailed in a report to the funding body^31^ and are summarised here.

**Figure 3:**
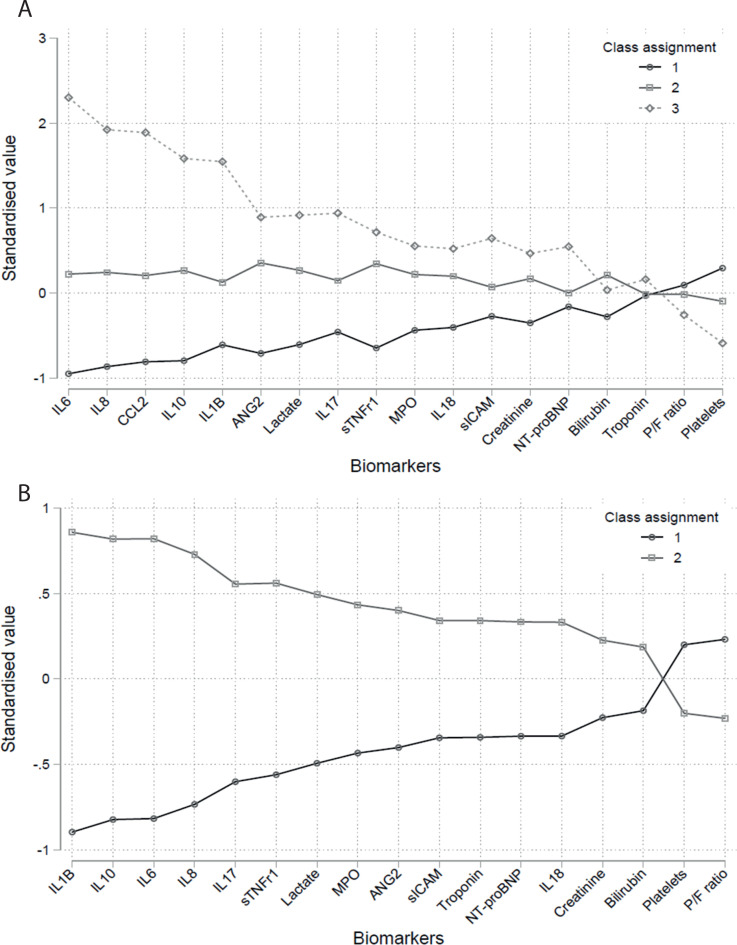
Estimated latent class analysis class means of each indicator by class for LeoPARDS (panel A) and VANISH (panel B) trials. Indicators have been standardised to ensure zero population mean and facilitate comparison. Indicators are ordered by the difference between classes for almost all indicators. In the LeoPARDS trial, indicators are ordered by the difference between class 1 and class 3, the estimated means for class 2 were in between those for classes 1 and 3. IL, interleukin; MPO, myeloperoxidase; P/F, PaO_2_:FiO_2_; sICAM, soluble intercellular adhesion molecule.

Latent class analysis found three classes in LeoPARDS (online supplemental tables S5–S7, online supplemental figure S3,^31^ which showed good concordance with hierarchical clustering derived groups (83%, online supplemental table S8). The variables showing the greatest separation between latent class analysis classes were IL-6, IL-8, MCP-1 (CCL2), IL-10 and IL-1β (figure 3, online supplemental table S9). As with hierarchical cluster analysis clusters, 28-day survival varied by class (p<0.001) and was lowest in class 3 (47%) compared with other classes (75%, class 1; 67%, class 2) (online supplemental table S10).

For VANISH, a two-class model was selected (online supplemental table S5, online supplemental figure S4.^31^ There was an even split between classes (90 individuals assigned to class 1 and 86 to class 2) and sensitivity analysis gave comparable results with 97% concordance (online supplemental table S7). Comparison of hierarchical cluster analysis cluster and latent class analysis class showed an overall concordance of 90% (online supplemental table S8). IL-1β, IL-10, IL-6 and IL-8 showed prominent class separation (figure 3, online supplemental table S9). Class 2 was associated with worse survival at 28 days (66% vs 80%, p=0.04) and more renal failure free days than class 2 (online supplemental table S10).

IL-6, IL-8, sTNFr1 and vasopressor use were available from our septic shock datasets, allowing us to fit two of the parsimonious ARDS models.^42^ The ARDS models classified the majority of patients as hyperinflamed (model 1 (IL-8, sTNFr1, vasopressors) 84% overall, 72% VANISH and 88% LeoPARDS; model 2 (IL-6, sTNFr1, vasopressors) 84% overall, 80% VANISH and 86% LeoPARDS) and showed limited concordance with our models (online supplemental table S11). In VANISH where both hierarchical clustering and latent class analysis suggested two groups, concordance between ARDS phenotypes and our latent or hierarchical class analysis-based models ranged from 69% to 77%.

### No evidence of heterogeneity of treatment effect based on inflammatory groupings

We found no significant heterogeneity of treatment effect using either hierarchical cluster or latent class analysis groupings for any of the outcomes tested (online supplemental table S12 and online supplemental figure S5). Similarly, we found no significant interaction between either of the ARDS model classifications and mortality for any of the trial treatments (p values for interaction 0.33–0.99).

### Grouping patients with sepsis based on leucocyte transcriptomics or plasma cytokine and related proteins shows association in the VANISH but not GAinS cohort

To understand how plasma cytokine-based patient clusters compare with our previously defined white blood cell transcriptomic clusters,^8 9^ we analysed cohorts where both data types were available (GAinS and VANISH). We found for the VANISH cohort, 63% of patients in the high cytokine group were also in SRS1, compared with 26% of patients in the low cytokine cluster (OR of being SRS1 if in the high vs low cytokine cluster: 4.72 (95% CI 2.3 to 9.5), p<0.0001, online supplemental table S4). Similarly, 60% of latent class analysis class 2 patients compared with 35% of class 1 had the SRS1 subphenotype (OR of being SRS1 in latent class analysis class 2 vs class 1: 2.8 (95% CI 1.5 to 5.3), p=0.001). However, there was no relationship between the plasma cytokine clusters and SRS in the GAinS cohort (p=0.51, online supplemental table S4). The lowest 28-day mortality was seen in those patients who had both SRS2 and low cytokine assignments (online supplemental figure S6).

Comparing plasma cytokines between SRS1 and SRS2, the pro-inflammatory cytokines IL-6, IL-8, MCP-1 and CCL3 were significantly higher in SRS1 than SRS2 (FDR <0.05) in both GAinS and VANISH (online supplemental table S13).

### Patient clusters derived from plasma cytokines show differences in gene expression

**Figure 4:**
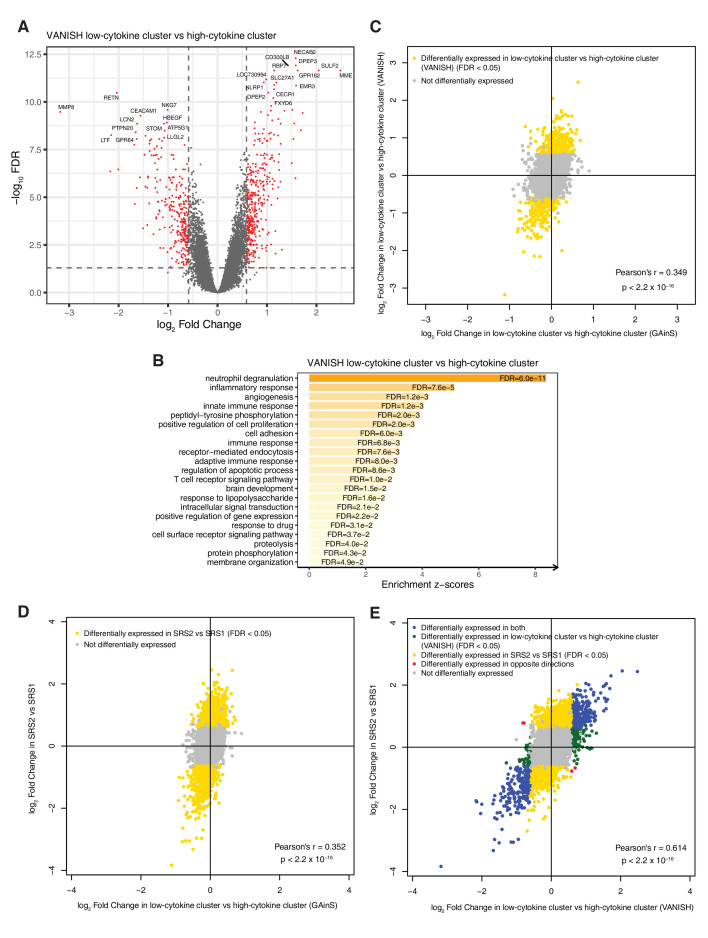
Gene expression comparisons between hierarchical cluster analysis inflammatory clusters, and correlation between inflammatory cluster comparisons and sepsis response signatures (SRS) transcriptomic subphenotype comparisons. (A) Volcano plot for low versus high cytokine clusters in VANISH and (B) the enriched Gene Ontology Biological Processes of the differentially expressed genes. (C) Log_2_ fold change correlations between the plasma cytokine cluster comparisons in VANISH and in Genomic Advances in Sepsis (GAinS). (D, E) Log_2_ fold change correlations of the inflammatory cluster comparisons and the SRS comparisons in GAinS (D) and in VANISH (E). P values are shown for tests of correlations using Pearson’s product moment correlation. In the volcano plot (A), red points indicate probes (n=667) for 559 differentially expressed genes (false discovery rate (FDR) <0.05 and fold change >1.5, dashed lines indicate these thresholds). Genes on the right-hand side have higher expression in the low cytokine cluster.

To better understand the connection between the inflammatory clusters across the cohorts, and the connection between these and the transcriptomic subphenotypes, we compared leucocyte gene expression between subgroupings. There were no significantly differentially expressed genes between inflammatory clusters in GAinS but 559 genes were differentially expressed (667 probes) in VANISH (FDR <0.05 and FC >1.5, figure 4). The latter were enriched for Gene Ontology Biological Process terms involving multiple aspects of the immune and inflammatory response (eg, ‘neutrophil degranulation’) as well as protein phosphorylation and angiogenesis (figure 4). In VANISH, there was a strong correlation between the fold changes in gene-expression between groups defined by hierarchical clustering or latent class analysis (Pearson’s r=0.950, p<2.2×10^−16^) suggesting both clustering approaches capture highly similar transcriptomic differences.

There was a statistically significant, though weak, correlation between the fold changes in gene-expression from the comparisons between the high cytokine and low cytokine clusters in GAinS or in VANISH (Pearson’s r=0.349, p<2.2×10^−16^, figure 4) suggesting that the inflammatory clusters in both datasets were capturing some of the same transcriptomic differences, although the magnitudes of difference observed were larger in VANISH.

Significant correlations were observed between differential gene expression in SRS subphenotypes and in cytokine clusters (figure 4), although this was weaker in GAinS than VANISH.

## Discussion

In this study, we have demonstrated that patients with sepsis show clustering based on plasma cytokine and related proteins into two or three distinct groups within which a high cytokine cluster that has the highest levels of individual proteins (pro-inflammatory and anti-inflammatory) had features of more severe disease and worse outcomes than the other patient clusters.

Perhaps the best recognised subphenotypes in critical illness are those described in ARDS, a common sequela of sepsis, where a two-group model has been described across several studies. High levels of IL-6, IL-8, sTNFr1 and low protein C discriminate a hyper-inflammatory subphenotype from a hypo-inflammatory one with worse survival and higher levels of shock in the hyper-inflammatory group.^21^ Although our data suggested similar inflammatory groups as described in ARDS with higher disease severity in the most hyperinflamed patients, when we fitted the parsimonious ARDS models to our data,^42^ we found that they allocated the majority of patients to the hyperinflamed group, much higher proportions than have been described in previous studies looking at these inflammatory subphenotypes in ARDS and patient populations at risk of but without ARDS.^16–18 20 21 43 44^ With such a high proportion of patients falling into one class, it raises doubts about the utility of the ARDS phenotypes in our septic shock cohorts and supports our approach of looking for subphenotypes more suitable for septic shock.

Both our data and the current literature support two-group^16–18 20 21 43^ and three-group^13 14^ inflammatory models in critical illness. The exact number of clusters is likely to vary depending on the variables used, the patient population, sample size and timing of blood sampling. Future studies with larger sample sizes and different sets of plasma cytokine and related proteins may discover novel groups. With these factors in mind, it is difficult to determine if plasma cytokine-derived groups defined across studies and patient populations are the same. However, across many studies in critical illness, including the one reported here, the identification of a group of patients with high plasma cytokines consistently shows prognostic enrichment for worse outcomes and disease severity such as incidence and severity of shock.^14 17 18 20 21^

Differential gene expression analysis gives some indication of biological processes that distinguished the high-cytokine from the low-cytokine clusters in our datasets, such as neutrophil degranulation and inflammatory response but it is likely that differences we observe reflect contributions from multiple cell and tissue types. In VANISH, neutrophil degranulation was the most enriched pathway, with no enrichment for individual cytokine pathways suggesting that cytokine clusters might originate from a global release of stored cytokines as opposed to upregulation or downregulation of individual cytokines. This could explain why pro-inflammatory and anti-inflammatory cytokines are at higher concentrations in the high cytokine group. This needs to be viewed as exploratory as we were unable to validate the findings in the GAinS cohort, possibly due to its limited sample size. Several of the genes that we found differentially expressed between our two cytokine groups were previously found to be differentially expressed between patients allocated to ARDS ‘reactive’ and ‘uninflamed’ biologic phenotypes.^45^ Of particular note are the MMP8 and MME genes which showed the greatest fold changes between phenotypes in both sets of analysis with MMP8 being overexpressed by the high inflammatory and MME by the low inflammatory phenotypes in both studies.

We were unable to identify heterogeneity of treatment effect between our plasma cytokine-based clusters and any of the trial therapies. This could be for a number of reasons. First, as a subgroup analysis of the original trial, we were not powered to identify differential treatment effect. Second, these may not be the optimal therapies to investigate, for example, norepinephrine, vasopressin and levosimendan are all cardiovascular drugs which have no or minimal effect on circulating cytokines.^46 47^ However, lack of heterogeneity of treatment effect in this study does not preclude plasma cytokine-based subphenotyping providing predictive enrichment for other treatments and highlights the importance of collecting biological samples alongside clinical trials to investigate these relationships.

At best, we found only a moderate relationship between SRS gene-expression endotypes and plasma cytokine clusters, which reflects the findings from a recently published study^48^ that also found limited overlap between clinical, inflammatory and gene-expression phenotypes. This study found a similar relationship between ARDS inflammatory phenotypes and SRS as we found between our sepsis cytokine clusters and SRS, likely reflecting different underlying mechanisms and processes contributing to the observed patient clusters. Further work is needed to understand the causal and temporal relationship between inflammation and transition into the SRS1 subphenotype with recent evidence, for example, of granulopoietic dysfunction involving specific neutrophil subsets.^49^ Based on the findings that transcriptomic and inflammatory phenotypes are not interchangeable and provide complementary biological information, future sepsis subphenotypes may benefit from combining both plasma protein and gene-expression data, with appropriately powered cohorts for validation designed to explore the extent to which subphenotypes exist within and across critical illness syndromes.^5^

There are limitations to this work. First, because of differences in patient cohorts, sample sizes, analytical platforms and panels of plasma proteins measured, it is impossible to be certain that the clusters and classes described are the same between patient cohorts. These differences may account for the fact that the values of some of the plasma proteins differ in each cluster from cohort to cohort and for some of the discrepancy in relationships with clinical outcomes between cytokine clusters in GAinS, VANISH and LeoPARDS. For example, the lack of differential gene expression between plasma cytokine-based clusters in GAinS may be a result of its small sample size. Similarly, different timing of sample collection in relationship to the onset of the inflammatory response and sepsis complicates interpretation of the relationship between cytokine and transcriptomic clustering across the datasets. There were also limitations associated with the platforms used to measure the cytokines. The multiplex system used in VANISH resulted in several assayed plasma proteins with concentrations below the level of quantification. Censoring of these data with the value of the lower limit would have introduced imprecision, compromising the interpretation of the importance of these plasma proteins. With the exception of LeoPARDS, the datasets are relatively small for cluster analysis which may explain why it was only in LeoPARDS that three clusters were identified. However, as a group of patients with the highest levels of plasma cytokine and related proteins had the worst disease severity across all datasets suggests that this finding was robust to this limitation. Finally, none of the trial datasets were designed with the intention of performing a subgroup analysis and as such are likely to be underpowered to detect a subgroup/treatment interaction. Therefore, it is important that these results are viewed as exploratory and not as conclusive evidence that no subphenotype/treatment interaction exists.

In conclusion, we identified clustering of patients with sepsis by plasma cytokine and related proteins, with high abundance clusters associated with worse disease severity and outcomes but no detectable heterogeneity in treatment effect in the two clinical trial cohorts. Future patient stratification, either for prognostication or for treatment allocation, may need to combine multiple types of biological data at scale to allow the most accurate classification. The exact composition of data types is likely to be based on the therapeutic decision that needs to be addressed.

## Data Availability

Data are available in a public, open access repository. Data are available on reasonable request. The gene expression data are available on ArrayExpress (https://www.ebi.ac.uk/biostudies/arrayexpress, accession: E-MTAB-4421/E-MTAB-4451/E-MTAB-5273/E-MTAB-5274/E-MTAB-7581). There are no conditions of reuse. Individual participant data that underlie the results in this article, after de-identification (text, table and figures) and biomarker data will be made available from the corresponding author on submission of a data request application.
