# Supplementary material for: Patient stratification using plasma cytokines and their regulators in sepsis: relationship to outcomes, treatment effect and leucocyte transcriptomic subphenotypes
Source: Thorax. 2024 Mar 12;79(6):515–23. doi: 10.1136/thorax-2023-220538 (PMC11137467; doi:10.1136/thorax-2023-220538)
Supplement: Supplementary data [file thorax-2023-220538supp001.pdf]

Supplementary Materials

Patient stratification using plasma cytokines and their regulators in sepsis: relationship to outcomes, treatment effect and leukocyte transcriptomic sub-phenotypes

David B Antcliffe, Yuxin Mi, Shalini Santhakumaran, Katie L Burnham, A Toby Prevost, Josie K Ward, Timothy Marshall, Claire Bradley, Farah Al-Beidh, Paula Hutton, Stuart McKechnie, Emma E Davenport, Charles J Hinds, Cecilia M O'Kane, Daniel F McAuley, Manu Shankar-Hari, Anthony C Gordon, Julian C Knight

Supplementary Methods

Table S1: Comparison of baseline variables and clinical outcomes between patient included and not included in hierarchical cluster analysis in VANISH and LeoPARDS

Table S2: Plasma cytokines and related proteins used for hierarchical cluster analysis in the three datasets

Table S3: Comparison of plasma cytokine and related protein concentrations between hierarchical clusters identified in the GAinS, VANISH and LeoPARDS datasets

Table S4: Comparison of baseline variables and clinical outcomes between clusters from the hierarchical cluster analysis models in GAinS, VANISH and LeoPARDS

Table S5: Fit statistics for latent class models

Table S6: Estimated class distribution, indicator means and separation for the LeoPARDS trial.

Table S7: Differences in class assignment for main and sensitivity analysis

Table S8: Confusion matrices comparing hierarchical cluster analysis cluster assignment and latent class for LeoPARDS and VANISH

Table S9: Latent class analysis variable data by class, with study participants assigned by highest posterior class probability in latent class analysis

Table S10: Baseline characteristics and outcomes by latent class analysis assigned class.

Table S11: Comparison of hierarchical cluster analysis and latent class analysis group membership with those derived from ARDS parsimonious models.

Table S12: Assessment of heterogeneity of treatment effect between hierarchical cluster analysis derived subgroups in VANISH and LeoPARDS.

Table S13: Differential plasma cytokine and related protein abundance analysis between the SRS1 and SRS2 transcriptomic sub-phenotypes in GAinS and VANISH.

Figure S1: Assessment of hierarchical clustering performance in GAinS, VANISH and LeoPARDS

Figure S2: Hierarchical clustering of natural log transformed plasma cytokines and related proteins in GAinS, VANISH and LeoPARDS

Figure S3: Plots of latent class analysis model fit indicators in the LeoPARDS trial

Figure S4: Plots of latent class analysis model fit indicators in the VANISH trial

Figure S5: Forrest plot showing the assessment of heterogeneity of treatment effect for the latent class analysis clusters in VANISH and LeoPARDS.

Figure S6: Comparison of 28-day mortality of patients in GAinS and VANISH stratified by SRS, cytokine clusters and a combination of the sub-phenotypes

Supplementary References

Supplementary Methods

The Vasopressin vs Noradrenaline as Initial Therapy in Septic Shock (VANISH) Trial

Design

VANISH was a double-blind, factorial (2x2), randomized controlled trial conducted in 18 intensive care units in the United Kingdom.

Treatment Regimen

Patients were randomized to receive blinded vasopressin (0-0·06 U/min) or noradrenaline (0-12 µg/min) as first line vasopressor to maintain the mean arterial blood pressure after adequate fluid resuscitation within the first 6 hours after onset of septic shock. If the maximal dose of the first study drug was reached patients were randomized to receive either hydrocortisone or placebo, the second study drug. If the patient was still hypotensive after the first dose of study drug 2 then open label catecholamine vasopressors could be used. If the patient was still not responding to open label vasopressors, then open label IV hydrocortisone could be given as a rescue therapy.

Blood sampling occurred in 10 centers where research nurses were available. The baseline sample was collected up to 12h after study drug 1 was administered, however, the majority were collected at or prior to drug administration.

Patients

Inclusion Criteria

Adult patients who required vasopressors for the management of sepsis despite adequate fluid resuscitation.

Patients needed to fulfil the following inclusion criteria:

- Fulfil 2/4 of the systemic inflammatory response syndrome criteria (1) due to known or suspected infection within the previous 24 hours.
- Hypotension despite adequate intravenous fluid resuscitation.

Exclusion Criteria

Patients were excluded if any of the following criteria were met:

- The patient had received a continuous infusion of vasopressors previously during the ICU admission (other than vasopressors used as emergency treatment [for less than six hours] to stabilize the patient during this episode). Vasopressors included noradrenaline, adrenaline, vasopressin, dopamine, metaraminol, phenylephrine, and (intermittent) terlipressin.
- Regular systemic corticosteroid therapy within the previous three months (this did not include inhaled steroid therapy).
- Known adrenal dysfunction / insufficiency.
- End-stage renal failure (i.e. requiring long term dialysis)
- The physician and team were not committed to full active care.
- The patient was known to be pregnant.
- The patient had known acute mesenteric ischemia.
- The patient was known to have Raynaud's phenomenon, systemic sclerosis or other vasospastic diseases.

- The patient had been enrolled in another clinical trial of an investigational medicinal product within 30 days or was enrolled in another interventional study that might interact with the study drugs.
- The patient had a history of anaphylaxis or hypersensitivity to any study drug.

Informed Consent

Due to the emergency nature of the trial, a waiver of initial consent was granted. Patients could be enrolled without prospective consent and then written consent was obtained from the patient or a personal or professional legal representative as soon as possible. For cases in which a legal representative gave consent, retrospective written consent was sought once the patient regained decision-making capacity.

Blood Sampling

Plasma was collected on the first day of septic shock. Blood samples were collected into 10mL EDTA vacutainers on ice, inverted 5-8 times and then centrifuged at 1000RCF for 10min before the plasma layer was aliquoted. Samples were separated locally, frozen, and sent to the coordinating center in batches for storage at -80°C and subsequent analysis.

Patient Selection for Current Study

One patient was excluded from the VANISH cohort due to uncertainty about the timing of the baseline blood sample and another because they had been incorrectly enrolled into the trial and had subsequently been excluded, so had no clinical data. For hierarchical clustering all patients were included where there was a complete set of baseline plasma protein data as this method is unable to handle missing data. Missing plasma protein data were due to technical issues so were considered missing at random.

The Levosimendan for the Prevention of Acute Organ Dysfunction in Sepsis (LeoPARDS) Trial

Design

LeoPARDS was a multicenter double-blind, placebo-controlled trial conducted in 34 Intensive Care Units (ICUs) in the United Kingdom between January 2014 and December 2015.

Treatment Regimen

Patients received levosimendan or placebo for 24 hours in addition to standard care. Drug infusion was started at 0.1 µg/kg per minute and increased after 2-4 hours to 0.2 µg/kg per minute for the remainder of the 24 hours. All other aspects of clinical care were at the discretion of the treating clinicians. Plasma samples were collected prior to randomization on the day of inclusion.

Patients

Inclusion Criteria

Adult patients with septic shock who required vasopressors for at least 4 hours and were recruited within 24 hours of meeting inclusion criteria were eligible for entry into the study. Inclusion criteria used the internationally established consensus definitions of sepsis at that time (2).

- Fulfil 2/4 of the criteria of the systemic inflammatory response syndrome

(SIRS) due to known or suspected infection within the previous 24 hours.

The SIRS criteria were:

(1) fever (>38 °C) or hypothermia (< 36 °C),

(2) tachycardia (heart rate > 90 beats per minute),

(3) tachypnoea (respiratory rate > 20 breaths per minute or PaCO2 < 4.3 kPa) or need for mechanical ventilation,

(4) abnormal leukocyte count (> 12,000 cells/mm3, < 4000 cells/mm3, or > 10 immature [band] forms).

• Hypotension, despite adequate intravenous fluid resuscitation, requiring treatment with a vasopressor infusion (e.g. noradrenaline / adrenaline / vasopressin analogue) for at least four hours and still having an ongoing vasopressor requirement at the time of randomization. Adequate fluid resuscitation was achieved using repeated fluid challenges.

Exclusion Criteria

• More than 24 hours since meeting all the inclusion criteria

• End-stage renal failure at presentation (previously dialysis-dependent)

• Severe chronic hepatic impairment (Child-Pugh class C)

• A history of Torsades de Pointes

• Known significant mechanical obstructions affecting ventricular filling or outflow or both.

• Treatment limitation decision in place (e.g, Do Not Attempt Resuscitation or not for ventilation/ dialysis)

• Known or estimated weight >135kg

• Known to be pregnant

• Previous treatment with levosimendan within 30 days

• Known hypersensitivity to levosimendan or any of the excipients

• Known to have received another investigational medicinal product within 30 days or currently in another interventional trial that might interact with the study drug.

Blood Sampling

Plasma was collected on the first day of septic shock. Samples were separated locally, frozen, and sent to the coordinating centre in batches for storage and subsequent analysis.

Genomic Advances in Sepsis (GAinS)

Design

Adult patients (>18y) were recruited at 23 UK ICUs between 2006 and 2015 as part of the UK Genomic Advances in Sepsis (GAinS) study (ukccggains.com) (3, 4). Ethics approval was granted nationally (REC Reference Number 05/MRE00/38 and 08/H0505/78) and for individual participating centers, with informed consent obtained from patients or their legal representative. Sepsis was diagnosed according to ACCP/SCCM guidelines, and all patients showed evidence of organ dysfunction (4). Community acquired pneumonia (CAP) was defined as a febrile illness associated with a cough, sputum production, breathlessness, leukocytosis and radiological features of pneumonia which was acquired in the community or within two days of ICU admission (5, 6). Fecal peritonitis (FP) was diagnosed at laparotomy as inflammation of the peritoneal membrane secondary to large bowel perforation and fecal contamination (7). Demographics and clinical covariates were

recorded using an electronic case report form which included details of the results of microbiological investigations as previously described (4).

Exclusion Criteria

Patient or legal representative unwilling or unable to give consent; age <18 years; pregnancy; advanced directive to withhold or withdraw life sustaining treatment; admission for palliative care only; or immune-compromise.

Blood Sampling

Blood samples were collected on the first day of ICU admission. For the plasma samples, blood was collected into one 5ml EDTA vacutainer, inverted gently 10 times, and then centrifuged at 1600RCF for 10min at 4◦C. 500µL aliquots of the plasma layer were then transferred into cryotubes and stored at -20◦C until being sent to the coordinating centre in batches for storage at -80◦C.

Plasma cytokine and related protein measurement

Sixty-five plasma proteins were measured in GAinS using the ProcartaPlex™ Luminex platform (ThermoFisher Scientific, Waltham, MA, USA), twenty-one in VANISH using the LEGENDplex™ multiplex assays (BioLegend, San Diego, USA) and enzyme linked immunosorbent assay (ELISA) ELLA™ multiplex assays (ProteinSimple, San Jose, CA, USA) (Supplementary table S2) and eleven in LeoPARDS using the enzyme linked immunosorbent assay (ELISA) ELLA™ multiplex assays (ProteinSimple, San Jose, CA, USA), ELLA™ Simple Plex assays (ProteinSimple, San Jose, CA, USA), Duoset ELISA (R&D Systems, Minneapolis, MN,

USA) assays (Supplementary table S2). All analysis was carried out as per the manufacturers' instructions for the assays used.

Hierarchical cluster analysis

Hierarchical cluster analysis was applied to the plasma protein panels in GAinS, VANISH and LeoPARDS independently. As hierarchical cluster analysis is unable to handle missing data and variables missing due to technical issues occurred at random, only patients with complete panels were included. Where measurements could be obtained but were above or below the limits of quantification these were censored at the quantification limit based on the standard curve for the analysis run. All plasma protein concentrations were natural log transformed prior to clustering. Dissimilarity between samples was measured by Euclidean distance. Ward's method was used as linkage for cluster agglomeration using the hclust function. We also deployed k-means clustering and consensus clustering (8) to help determine the optimal number of clusters and assess reproducibility. For each cluster number from two to ten, k-means clustering with a ten-fold cross-validation was calculated on 90% of the samples each time and testing on the remaining 10% over 60 repetitions. Consensus clustering was performed over 1000 iterations of the hierarchical cluster analysis process described above, in each of which 80% of the subjects and 95% of the plasma proteins were randomly sampled for inclusion, using the ConsensusClusterPlus package (9). The optimal number of clusters was defined by inspection of the dendrograms, the drop of test sample distances to cluster centres in k-means, and the cumulative distribution of the consensus index in each cluster number. Another approach that we took to remove the subjectivity of deciding on the optimal number of clusters was to use the NbClust package

that applies twenty-six metrics to determine the optimal number of clusters (10). Clustering was performed in R (11) and the final cluster assignments for further analysis were derived using the hclust function as these are highly concordant with the consensus clusters but with a more accessible repeatable algorithm and clear interpretations.

Latent class analysis

Latent class analysis is used to estimate a latent (i.e. unobserved) categorical variable which assigns individuals to groups (*classes*), when we have a set of observed data (*indicators*) which we believe is distributed differently for each class. Latent class analysis is a type of finite mixture model that jointly estimates a model for each of the indicators. Simultaneously, a multinomial logistic model for probabilities of class membership is estimated. The number of classes is specified in the model, but models with different numbers of classes can be compared.

Latent class analysis has previously been caried out independently on the LeoPARDS and VANISH datasets and is described in full in a report to the funding body that has supported some of the work (12). Briefly, latent class analysis analyses were carried out separately for LeoPARDS and VANISH cohorts, agnostic of outcome, as both had the same plasma proteins and clinical variables available. Clinical and inflammatory plasma proteins were chosen for inclusion in the latent class analysis models based on their associations with sepsis pathophysiology (PaO_2/FiO_2 ratio, creatinine, platelets, bilirubin, IL-1β, IL-6, IL-8, IL-10, IL-17, IL-18, MPO, sICAM, ANG-2, troponin, NT-proBNP, sTNFr, lactate and MCP-1 (CCL2)). Other baseline clinical and demographic variables (age, ethnicity, BMI, co-morbidities (any of NYHA class IV, severe COPD, chronic renal failure, cirrhosis, immunodeficiency), site of

infection (lung/abdomen/urine/other), SOFA score, the acute physiology element of the APACHE II score (APS-AP II), and post-surgical admission) which may be predictive of subclass were included in the model as class predictors. Normal distributions were used for the continuous indicators, applying natural log transformations as necessary. All variables were standardized to have a mean of 0 and standard deviation of 1, with parameters taken from the data within the limits of detection. If a patient has any missing individual indicators, latent class analysis still allows the rest of the complete data to be included, implicitly making the assumption that the data are missing at random (i.e. the probability of missingness depends only on the observed data and not any missing data). This is reasonable for the plasma protein data as missing individual indicators were due to technical issues.

Latent class analysis models were fit in three stages. First conditional independence was assumed (all covariances constrained to zero) and no covariates predicting class membership were included. Secondly, pre-specified clinical and demographic variables measured at baseline were included as covariates predicting class membership. Thirdly, variance assumptions concerning indicators were relaxed to allow (a) non-constant residual variance across classes (b) non-zero covariances (c) both of these. It was not possible within the software used to model covariances between censored variables. For each stage we first fit a 1 –class model, then increased the number of classes by 1 until convergence could not be achieved. A number of strategies were used to achieve convergence, namely: (i) for a k-class model, using starting values from a k-1 class model (ii) using alternative integration methods (iii) reducing the number of censored indicators by treating values outside the limits as having values equal to the limit, for indicators with less than 5 such values (iv)

reducing the number of class predictors, selecting covariates which improved model fit based on likelihood ratio tests.

For each latent class analysis model, the class means were estimated and differences across classes compared to determine which indicators showed the most separation across classes. For each model and each participant, the probability of an individual being in each class was predicted, with the probabilities for a participant summing to 1 across the classes. Each participant could then be assigned to the class with which they have the highest class probability. The Bayesian Information Criterion (BIC) was the primary measure of model selection, with smaller values indicating better fit. We also considered the Akaike Information Criterion (AIC), log likelihood, entropy (a measure of class separation between 0 and 1) (13), class sizes (with very small classes being indicative of overfitting) and the mean probability of class assignment, averaged over participants in the class. We also assessed the class means and sizes to see if the substantive interpretation of the classes differed across models and plots of the change in fit statistics with the number of classes were used to determine where additional classes gave limited improvement in fit (14).

Sensitivity analysis

In the main analysis we drew a distinction between class-defining variables (Indicators) and class-predicting variables. As a sensitivity analysis we compared the class groupings when including all variables as indicators in the latent class model, following earlier work by Calfee and colleagues (15–19).

Transcriptomic sub-phenotypes and differential gene expression

Genome-wide transcriptomic data in blood leukocytes, acquired using Illumina Human-HT-

12 v4 Expression BeadChips (47,323 probes), were available from the VANISH and GAinS studies as described previously (4, 20, 21). Raw data were prepared using Illumina's Genomestudio. Background signals were subtracted and probes with a detection p-value <0·05 in at least 10% of samples were retained for downstream analysis. SRS sub-phenotypes have previously been assigned in the GAinS patients using unsupervised clustering on genome-wide transcriptomic data or a predictive 7-gene model, and in VANISH patients using the 7-gene model (4, 20, 21).

Microarray data from GAinS and VANISH were co-normalized using the vsn package (22) and batch corrected using the ComBat function from the sva package (23) in R, resulting in 28220 communal probes after quality control. Following additional quality control with MixupMapper (24), baseline gene expression data were available for 115 patients in GAinS and 149 in VANISH from those included in the plasma cytokine and related protein hierarchical cluster analysis. Differentially expressed genes between sub-phenotypes were identified using the limma package (25), which fits a linear model to the expression of each gene and applies an empirical Bayes smoothing to the standard errors of the estimated log-fold changes to account for the overall variance. For a gene to be differentially expressed it had to both meet the false discovery rate (FDR) threshold of <0.05 and a fold change >1.5 in accordance with previous work (4, 21). Pathway enrichment analysis was performed with the R package XGR (26), using annotations of Gene Ontology Biological Process. The differentially expressed genes were tested against the background of all genes with a hypergeometric distribution. Contrasts between the plasma protein clusters or SRS groups were limited to the same subsets of patients with both assignments available.

Supplementary Tables:

Table S1: Comparison of baseline variables and clinical outcomes for patients included for hierarchical cluster analysis from the VANISH and LeoPARDS trials and those not included. Continuous variables have been compared with the Mann-Whitney U test and categorical variables with the chi-squared test. P-values in bold are those <0.05. No adjustment has been made for multiple comparisons. (APACHE=Acute Physiology and Chronic Health Evaluation, COPD=Chronic Obstructive Pulmonary Disease, GCS=Glasgow Coma Scale, IV=intravenous, IQR=interquartile range). * Organ failure was defined as having a Sequential Organ Failure Assessment (SOFA) score of 3 or more and acute renal failure was defined as having acute kidney injury stage 3.

	**VANISH**			**LeoPARDS**		
	**Patients Included in Hierarchical Cluster Analysis**	**Patients Not Included in Hierarchical Cluster Analysis**	**p-value**	**Patients Included in Hierarchical Cluster Analysis**	**Patients Not Included in Hierarchical Cluster Analysis**	**p-value**
Age, median (IQR), years	65 (54-77)	66 (55.5-77)	0.78	68 (58-76)	69 (53-76)	0.73
Males, No./total (%)	101/155 (65)	137/254 (54)	**0.03**	268/484 (55)	21/31 (68)	0.18
Caucasian, No./total (%)	128/155 (83)	221/254 (87)	0.22	452/484 (93)	28/31 (90)	0.51
Weight, median (IQR), kg	75 (62-90)	74 (63-85)	0.29	78 (66-90)	80 (65-90)	0.60
BMI, median (IQR), kg/m2	26 (22-32)	25 (22-29)	0.27	27 (23-31)	27 (21-32)	0.70
Recent surgical history, No./total (%)	25/155 (16)	48/254 (19)	0.48	176/484 (36)	13/31 (42)	0.53
APACHE II score, median (IQR)	23 (19-30)	24 (19-30)	0.99	25 (21-31)	24 (17-31)	0.31
Randomised to Vasopressin, No./total (%)	78/155 (50)	127/254 (50)	0.95	-	-	-
Randomised to Hydrocortisone, No./total (%)	78/155 (50)	124/254 (49)	0.77	-	-	-
Randomised to Levosimendan, No./total (%)	-	-	-	243/484 (50)	15/31 (48)	0.84
Pre-existing conditions						
Ischaemic heart disease, No./total (%)	25/155 (16)	37/254 (15)	0.67	71/484 (15)	6/31 (19)	0.48
Severe COPD, No./total (%)	8/155 (5)	7/254 (3)	0.21	23/484 (5)	4/31 (13)	0.05
Chronic renal failure, No./total (%)	7/155 (5)	20/254 (8)	0.18	35/484 (7)	2/31 (6)	0.87
Cirrhosis, No./total (%)	10/155 (6)	5/254 (2)	**0.02**	9/484 (2)	1/31 (3)	0.59
Cancer, No./total (%)	18/155 (12)	29/254 (11)	0.95	-	-	-
Immunocompromise, No./total (%)	10/155 (6)	18/254 (7)	0.81	44/484 (9)	3/31 (10)	0.91
Diabetes, No./total (%)	31/155 (20)	59/254 (23)	0.44	104/484 (21)	6/31 (19)	0.78
*Organ Failure**						
Respiratory failure, No./total (%)	56/153 (37)	93/249 (37)	0.88	187/482 (39)	13/31 (42)	0.73
Renal failure, No./total (%)	35/155 (23)	50/253 (20)	0.50	51/482 (11)	6/31 (19)	0.13
Liver failure, No./total (%)	10/137 (7)	10/242 (4)	0.19	13/474 (3)	1/30 (3)	0.85
Haematological failure, No./total (%)	9/151 (6)	13/247 (5)	0.77	26/481 (5)	3/30 (10)	0.29
Neurological failure, No./total (%)	50/149 (34)	78/236 (33)	0.92	214/410 (52)	14/26 (54)	0.87
Physiological Variables						
Mean arterial pressure, median (IQR), mmHg	69 (61.75-76)	70 (62-78)	0.36	74 (68-79)	75 (65-80)	0.93

	**VANISH**			**LeoPARDS**		
	**Patients Included in Hierarchical Cluster Analysis**	**Patients Not Included in Hierarchical Cluster Analysis**	**p-value**	**Patients Included in Hierarchical Cluster Analysis**	**Patients Not Included in Hierarchical Cluster Analysis**	**p-value**
Heart rate, median (IQR), beats per minute	95 (81-110)	99 (86-110)	0.11	95 (80-111)	96 (83-110)	0.53
Central Venous Pressure, median (IQR), mmHg	13 (9-19)	12 (9-16)	0.10	11 (8-15)	11 (8-14)	0.27
Lactate, median (IQR), mmol/L	2.4 (1.5-4.45)	2.3 (1.3-3.875)	0.30	2.2 (1.4-3.5)	3.3 (1.8-6.7)	**0.04**
paO2:fiO2 ratio, median (IQR), kPa	26 (17-42)	25 (16-39)	0.30	29 (20-39)	28 (19-39)	0.47
Creatinine, median (IQR), mmol/L	119 (77-189)	125.5 (73.25-209.75)	0.82	138 (91-213)	138 (105-218)	0.42
Bilirubin, median (IQR), mmol/L	15 (9-28)	15 (8-27)	0.72	14 (8-26)	15 (9-30)	0.49
Platelet count, median (IQR), x10^3/L	188 (121-298)	184 (121-283)	0.68	216 (144-308)	170 (101-266)	0.06
GCS, median (IQR)	14 (4.5-15)	14 (3.25-15)	0.73	9 (3-15)	7 (3-15)	0.68
Mechanical ventilation, No./total (%)	82/155 (53)	154/254 (61)	0.12	390/484 (81)	27/31 (87)	0.37
Renal replacement therapy, No./total (%)	5/155 (3)	6/254 (2)	0.60	86/484 (18)	3/31 (10)	0.25
Volume of IV fluid in previous 4 h, median (IQR), mL	1056 (539.75-1964)	1170 (750-2081)	0.07	728 (438-1202)	896 (551-1701)	0.12
Dose of noradrenaline at randomisation, median (IQR), ug/kg/min	0.15 (0.09-0.25)	0.18 (0.11-0.36)	**0.03**	0.28 (0.16-0.47)	0.36 (0.18-0.5)	0.57
Time from onset of shock to randomisation, median (IQR), hours	4 (2-5)	3 (1-5)	0.27	16 (10-21)	15 (8-22)	0.60
Source of Infection						
Lung, No./total (%)	65/152 (43)	100/248 (40)	0.63	189/484 (39)	12/31 (39)	0.97
Abdomen, No./total (%)	33/152 (22)	60/248 (24)	0.57	177/484 (37)	14/31 (45)	0.34
Soft tissue, No./total (%)	5/152 (3)	14/248 (6)	0.28	25/484 (5)	1/31 (3)	0.63
Other, No./total (%)	49/152 (32)	74/248 (30)	0.61	93/484 (19)	4/31 (13)	0.38
28-day mortality, No./total (%)	44/155 (28)	75/253 (30)	0.79	155/483 (32)	13/31 (42)	0.26

Table S2: List of plasma cytokines and related proteins measured in the three data sets and used for hierarchical cluster analysis with the percentage of samples above and below the limits of quantification. LLOQ, lower limit of quantification; ULOQ, upper limit of quantification.

	GAinS (n=124)			VANISH (n=155)			LeoPARDS (n=484)		
Analyte	Percentage <LLOQ	Percentage >ULOQ	Platform	Percentage <LLOQ	Percentage >ULOQ	Platform	Percentage <LLOQ	Percentage >ULOQ	Platform
MCP-1	0.0	0.0	Luminex	0.0	2.6	LEGENDplex™	0.0	1.0	ELISA
IL-6	0.0	3.2	Luminex	0.0	13.5	LEGENDplex™	0.0	6.6	ELISA
IL-8	1.6	0.0	Luminex	10.3	1.3	LEGENDplex™	0.0	0.6	ELISA
IL-10	0.8	0.0	Luminex	9.7	0.0	LEGENDplex™	0.0	0.2	ELISA
IL-18	0.0	0.0	Luminex	1.9	0.0	LEGENDplex™	0.8	2.5	ELISA
CCL3	1.6	0.0	Luminex	25.8	0.0	LEGENDplex™	-	-	-
IP-10	0.0	32.3	Luminex	1.9	0.6	LEGENDplex™	-	-	-
IFN-γ	0.0	1.6	Luminex	55.5	0.6	LEGENDplex™	-	-	-
IL-1β	3.2	0.0	Luminex	67.1	0.0	LEGENDplex™	8.9	0.0	ELISA
IL-2	26.6	0.0	Luminex	66.5	0.0	LEGENDplex™	-	-	-
IL-17	0.0	0.0	Luminex	66.5	0.0	LEGENDplex™	1.9	0.0	ELISA
TNF-α	0.0	0.0	Luminex	78.1	0.0	LEGENDplex™	-	-	-
IFN-α	36.3	0.0	Luminex	71.6	0.0	LEGENDplex™	-	-	-
IL-12p70	3.2	0.0	Luminex	88.4	0.0	LEGENDplex™	-	-	-
IL-4	0.0	0.0	Luminex	87.7	0.0	LEGENDplex™	-	-	-
IL-1α	13.7	0.8	Luminex	77.4	0.0	LEGENDplex™	-	-	-
MCP-2	0.0	0.8	Luminex	-	-	-	-	-	-
IL-2R	1.6	0.8	Luminex	-	-	-	-	-	-
SDF-1α	0.0	3.2	Luminex	-	-	-	-	-	-
IL-27	0.0	0.0	Luminex	-	-	-	-	-	-
LIF	5.6	0.0	Luminex	-	-	-	-	-	-

	GAinS (n=124)			VANISH (n=155)			LeoPARDS (n=484)		
Analyte	Percentage <LLOQ	Percentage >ULOQ	Platform	Percentage <LLOQ	Percentage >ULOQ	Platform	Percentage <LLOQ	Percentage >ULOQ	Platform
IL-5	6.5	0.0	Luminex	-	-	-	-	-	-
IL-7	0.0	0.0	Luminex	-	-	-	-	-	-
BLC	0.0	0.0	Luminex	-	-	-	-	-	-
Eotaxin-2	4.0	0.0	Luminex	-	-	-	-	-	-
Eotaxin	0.0	0.0	Luminex	-	-	-	-	-	-
IL-13	0.0	0.0	Luminex	-	-	-	-	-	-
IL-31	16.9	0.0	Luminex	-	-	-	-	-	-
SCF	0.0	0.0	Luminex	-	-	-	-	-	-
G-CSF	20.2	2.4	Luminex	-	-	-	-	-	-
GM-CSF	0.0	0.0	Luminex	-	-	-	-	-	-
HGF	0.0	5.6	Luminex	-	-	-	-	-	-
MIP-1β	0.8	0.0	Luminex	-	-	-	-	-	-
Eotaxin-3	0.0	0.0	Luminex	-	-	-	-	-	-
IL-9	0.0	0.0	Luminex	-	-	-	-	-	-
MIF	0.0	0.8	Luminex	-	-	-	-	-	-
TNF-β	4.0	0.0	Luminex	-	-	-	-	-	-
bNGF	0.8	0.0	Luminex	-	-	-	-	-	-
MIP-3α	0.0	0.0	Luminex	-	-	-	-	-	-
I-TAC	0.0	0.0	Luminex	-	-	-	-	-	-
TRAIL	0.0	0.0	Luminex	-	-	-	-	-	-
Fractalkine	4.8	0.0	Luminex	-	-	-	-	-	-
GRO-α	3.2	0.8	Luminex	-	-	-	-	-	-
IL-23	0.0	0.0	Luminex	-	-	-	-	-	-
MMP-1	4.0	0.0	Luminex	-	-	-	-	-	-
IL-15	31.5	0.0	Luminex	-	-	-	-	-	-

	GAinS (n=124)			VANISH (n=155)			LeoPARDS (n=484)		
Analyte	Percentage <LLOQ	Percentage >ULOQ	Platform	Analyte	Percentage <LLOQ	Percentage >ULOQ	Platform	Analyte	Percentage <LLOQ
M-CSF	0.0	12.1	Luminex	-	-	-	-	-	-
MCP-3	0.8	0.0	Luminex	-	-	-	-	-	-
MIG	1.6	0.0	Luminex	-	-	-	-	-	-
IL-16	0.8	0.0	Luminex	-	-	-	-	-	-
IL-21	0.8	0.0	Luminex	-	-	-	-	-	-
IL-3	4.8	0.0	Luminex	-	-	-	-	-	-
CD40-ligand	0.0	0.0	Luminex	-	-	-	-	-	-
FGF-2	3.2	0.0	Luminex	-	-	-	-	-	-
IL-22	2.4	0.8	Luminex	-	-	-	-	-	-
VEGF-A	1.6	0.0	Luminex	-	-	-	-	-	-
TSLP	0.0	0.0	Luminex	-	-	-	-	-	-
IL-20	0.0	0.0	Luminex	-	-	-	-	-	-
ENA-78	0.0	0.0	Luminex	-	-	-	-	-	-
CD30	0.0	0.0	Luminex	-	-	-	-	-	-
TNF-RII	0.0	0.0	Luminex	-	-	-	-	-	-
BAFF	7.3	0.0	Luminex	-	-	-	-	-	-
MDC	0.0	0.0	Luminex	-	-	-	-	-	-
APRIL	0.0	0.0	Luminex	-	-	-	-	-	-
Tweak	0.0	0.0	Luminex	-	-	-	-	-	-
ICAM	-	-	-	3.9	0.0	ELISA	0.2	4.5	ELISA
CCL5	-	-	-	7.1	1.3	LEGENDplex™	-	-	-
sTNFR	-	-	-	0.0	1.3	ELISA	0.0	0.0	ELISA
MPO	-	-	-	0.6	1.3	ELISA	7.2	2.5	ELISA
Ang-2	-	-	-	1.3	0.0	ELISA	1.4	1.7	ELISA

Table S3. Comparison of plasma cytokine and related protein concentrations between hierarchical clusters identified in the GAinS, VANISH and LeoPARDS datasets. Data are given as median and interquartile range in units of pg/mL and comparison has been made with the Mann-Whitney U test for two-group comparisons and the Kruskal-Wallis tests for three-group comparisons. FDR values in bold are those <0.05.

	**GAinS**			**VANISH**			**LeoPARDS**			
Analyte	Low cytokine cluster	High cytokine cluster	FDR	Low cytokine cluster	High cytokine cluster	FDR	Low cytokine cluster	Intermediate cytokine cluster	High cytokine cluster	FDR
n	70	54	-	71	84	-	191	208	85	-
MCP-1 (CCL2)	231.6 (124.2-402.6)	444.1 (285.7-874.3)	**<1x10^{-5}**	890.1 (533.9-1477.5)	4410.2 (2061.5-9789.2)	**<1x10^{-10}**	390.3 (279.5-623.9)	867.2 (640.1-1333.3)	3236.6 (1944-5282.3)	**<1x10^{-50}**
IL-6	980.3 (726.9-1516.6)	1768.5 (1384.7-3005.1)	**<1x10^{-5}**	337.4 (163.2-941.8)	11535.1 (3394.2-34623.2)	**<1X10^{-10}**	266.5 (100.4-575.6)	1089.3 (434.3-3169.8)	24761.5 (9608.8-40000)	**<1x10^{-50}**
IL-8	314.1 (187.7-411.5)	624.4 (515-1052.1)	**<1x10^{-10}**	32.1 (7.7-95.0)	839.8 (186.3-3192.8)	**<1X10^{-10}**	48.4 (30.4-91.6)	226 (138.7-375.2)	1931.8 (787-5332.2)	**<1x10^{-50}**
IL-10	156.9 (91.7-242.2)	359.7 (239.3-701.9)	**<1x10^{-10}**	10.2 (4.2-27.1)	151.4 (44.7-397.5)	**<1x10^{-10}**	25.4 (16.7-43)	112.5 (70-196.9)	432.2 (203.7-1049.9)	**<1x10^{-50}**
IL-18	607.5 (483.8-877.5)	1173.5 (999.9-1551.3)	**<1x10^{-10}**	354.3 (148.1-595.2)	536.9 (335.7-823.4)	**0.0003**	577.6 (381.3-905.3)	912.5 (567.9-1363.3)	896 (556.9-1642.5)	**<1x10^{-5}**
CCL3	72.1 (60-97.4)	138.3 (108.6-176.9)	**<1x10^{-10}**	11.9 (3.8-22.5)	79.4 (32.0-144.0)	**<1x10^{-10}**	-	-	-	-
IP-10	332.6 (234.8-544.4)	784 (409.5-787.5)	**<1x10^{-05}**	443.3 (214.1-783.4)	1248.6 (584.8-5498.9)	**<1x10^{-5}**	-	-	-	-
IFN-γ	487.7 (385.9-609.2)	977.5 (741.7-1520.9)	**<1x10^{-10}**	3.6 (2.3-13.2)	11.4 (3.6-175.0)	**0.0002**	-	-	-	-
IL-1β	28 (12.7-65.3)	151.7 (71.8-282.6)	**<1x10^{-10}**	3.2 (2.8-3.8)	3.8 (3.0-10.7)	**0.001**	0.8 (0.5-1.3)	1.4 (0.7-2.6)	4.6 (1.9-10.3)	**<1x10^{-20}**
IL-2	24 (22-31.8)	42.6 (32-59.5)	**<1x10^{-05}**	4.5 (3.3-9.5)	8.2 (4.5-14.5)	**0.02**	-	-	-	-
IL-17	488 (330.2-646.7)	846.4 (699.9-1147.5)	**<1x10^{-05}**	4.6 (2.7-7.7)	6 (4.4-17.7)	**0.03**	6.6 (4.6-9.7)	9.6 (6.1-19)	18 (7.7-48)	**<1x10^{-10}**
TNF-α	56.2 (34.5-82.4)	138.2 (104.3-187)	**<1x10^{-10}**	3.1 (2.4-5.3)	3.9 (3.0-6.7)	**0.03**	-	-	-	-

	GAinS			VANISH			LeoPARDS			
Analyte	Low cytokine cluster	High cytokine cluster	FDR	Low cytokine cluster	High cytokine cluster	FDR	Low cytokine cluster	Intermediate cytokine cluster	High cytokine cluster	FDR
IFN-α	75 (75-104.6)	172.9 (130.6-229.6)	**$<1x10^{-10}$**	3.6 (2.8-4.6)	3.9 (2.9-6.9)	0.07	-	-	-	-
IL-12p70	49.1 (24.7-75.1)	151.7 (111.6-225)	**$<1x10^{-10}$**	2.9 (2.4-4.4)	3.7 (2.9-5.2)	0.1	-	-	-	-
IL-4	1856.2 (1402.9-2561.8)	3319.6 (2659.1-4507.2)	**$<1x10^{-05}$**	2.6 (2.5-5.4)	4.9 (2.6-5.7)	0.18	-	-	-	-
IL-1α	46.3 (27-63.3)	153.8 (106.5-188.7)	**$<1x10^{-10}$**	4.8 (2.8-15.9)	5.3 (3.9-15.6)	0.52	-	-	-	-
MCP-2	76 (47.1-107.3)	197.9 (129.8-291.5)	**$<1x10^{-10}$**	-	-	-	-	-	-	-
IL-2R	12040.3 (7097.4-27527.9)	29087.4 (18754.9-63393.4)	**$<1x10^{-05}$**	-	-	-	-	-	-	-
SDF-1α	1761.3 (1212.4-2721.2)	5347.9 (3486.1-7304.9)	**$<1x10^{-10}$**	-	-	-	-	-	-	-
IL-27	1192.6 (838.4-1707.3)	2492.1 (1744.7-3450)	**$<1x10^{-10}$**	-	-	-	-	-	-	-
LIF	11.2 (6.3-18.7)	27.9 (19.3-46.8)	**$<1x10^{-10}$**	-	-	-	-	-	-	-
IL-5	58.5 (36.4-104.1)	247 (175.7-343.4)	**$<1x10^{-10}$**	-	-	-	-	-	-	-
IL-7	19.5 (9.5-34.4)	48.9 (34.6-78.7)	**$<1x10^{-5}$**	-	-	-	-	-	-	-
BLC	255.5 (161.6-527.5)	669.5 (373.8-1316.5)	**$<1x10^{-5}$**	-	-	-	-	-	-	-
Eotaxin-2	187.6 (126.4-278.1)	371.3 (282.9-611.4)	**$<1x10^{-5}$**	-	-	-	-	-	-	-

	**GAinS**			**VANISH**			**LeoPARDS**			
Analyte	Low cytokine cluster	High cytokine cluster	FDR	Low cytokine cluster	High cytokine cluster	FDR	Low cytokine cluster	Intermediate cytokine cluster	High cytokine cluster	FDR
Eotaxin	96.4 (68.1-110.8)	143.2 (114.4-186.8)	**<1x10^{-10}**	-	-	-	-	-	-	-
IL-13	95.9 (69.6-135.3)	178.5 (154.4-251.1)	**<1x10^{-10}**	-	-	-	-	-	-	-
IL-31	585.5 (306.2-1033.1)	1391.3 (889.9-2566.7)	**<1x10^{-05}**	-	-	-	-	-	-	-
SCF	55.1 (37.4-74.3)	137.3 (100.4-195)	**<1x10^{-10}**	-	-	-	-	-	-	-
G-CSF	79.6 (39.5-160)	323.5 (210.8-616.9)	**<1x10^{-10}**	-	-	-	-	-	-	-
GM-CSF	162.7 (91.4-243.4)	465.1 (309-621)	**<1x10^{-10}**	-	-	-	-	-	-	-
HGF	1405.2 (1041.3-2311.3)	2910.4 (1938.4-7442.3)	**<1x10^{-05}**	-	-	-	-	-	-	-
MIP-1β	174.6 (123.7-258.1)	375.5 (298.4-731.8)	**<1x10^{-10}**	-	-	-	-	-	-	-
Eotaxin-3	92.9 (67.9-120.7)	176.3 (133.3-226.9)	**<1x10^{-10}**	-	-	-	-	-	-	-
IL-9	163.4 (117.3-229.8)	411.7 (315.5-555)	**<1x10^{-10}**	-	-	-	-	-	-	-
MIF	19.9 (13.1-29.6)	39.9 (29.5-58.8)	**<1x10^{-5}**	-	-	-	-	-	-	-
TNF-β	337.2 (207.6-560.3)	986.9 (729.6-1537.1)	**<1x10^{-10}**	-	-	-	-	-	-	-
bNGF	805.6 (655.3-963.6)	1177.4 (1035.5-1468.6)	**<1x10^{-5}**	-	-	-	-	-	-	-
MIP-3α	801.8 (475.8-1308)	1670.3 (1235.2-2902.5)	**<1x10^{-5}**	-	-	-	-	-	-	-
I-TAC	319.5 (220.5-488.3)	845.9 (633.2-1306.5)	**<1x10^{-10}**	-	-	-	-	-	-	-

	**GAinS**			**VANISH**			**LeoPARDS**			
Analyte	Low cytokine cluster	High cytokine cluster	FDR	Low cytokine cluster	High cytokine cluster	FDR	Low cytokine cluster	Intermediate cytokine cluster	High cytokine cluster	FDR
TRAIL	128.4 (90.9-167.3)	298.1 (228-404.5)	**$<1x10^{-10}$**	-	-	-	-	-	-	-
Fractalkine	13.5 (6.6-17.1)	37.5 (28.7-56.7)	**$<1x10^{-10}$**	-	-	-	-	-	-	-
GRO-α	71.6 (29.2-101)	187.6 (143.1-255.9)	**$<1x10^{-10}$**	-	-	-	-	-	-	-
IL-23	1712.9 (1172.1-2420.7)	3119.8 (2392-3993.7)	**$<1x10^{-10}$**	-	-	-	-	-	-	-
MMP-1	51.5 (28.4-86.1)	135 (101.9-215.8)	**$<1x10^{-10}$**	-	-	-	-	-	-	-
IL-15	13.4 (13.4-29.2)	64.8 (42.2-108.3)	**$<1x10^{-10}$**	-	-	-	-	-	-	-
M-CSF	1232.2 (767.4-1662.8)	3026.1 (2198.1-4189.1)	**$<1x10^{-10}$**	-	-	-	-	-	-	-
MCP-3	184.3 (124.1-251.8)	446.7 (329-618)	**$<1x10^{-10}$**	-	-	-	-	-	-	-
MIG	99.4 (64.2-170.2)	401.1 (245.3-769.5)	**$<1x10^{-10}$**	-	-	-	-	-	-	-
IL-16	584.4 (363.6-820.8)	990.7 (670.7-1328.2)	**$<1x10^{-5}$**	-	-	-	-	-	-	-
IL-21	149.5 (72.9-213.1)	362.9 (277.8-518.3)	**$<1x10^{-10}$**	-	-	-	-	-	-	-
IL-3	93.6 (64-138.4)	398.7 (254.7-628.3)	**$<1x10^{-10}$**	-	-	-	-	-	-	-
CD40-ligand	170.4 (128-266.5)	467.2 (315.5-644)	**$<1x10^{-10}$**	-	-	-	-	-	-	-
FGF-2	293 (214.6-430.1)	665.9 (547.9-808.1)	**$<1x10^{-10}$**	-	-	-	-	-	-	-
IL-22	389.8 (262.9-517.9)	1121 (801.5-1551.4)	**$<1x10^{-10}$**	-	-	-	-	-	-	-

	GAinS			VANISH			LeoPARDS			
Analyte	Low cytokine cluster	High cytokine cluster	FDR	Low cytokine cluster	High cytokine cluster	FDR	Low cytokine cluster	Intermediate cytokine cluster	High cytokine cluster	FDR
VEGF-A	326.3 (237.9-501.1)	888.3 (696.3-1294.5)	**$<1x10^{-10}$**	-	-	-	-	-	-	-
TSLP	132.7 (97.7-185.5)	268.1 (220.9-416.9)	**$<1x10^{-10}$**	-	-	-	-	-	-	-
IL-20	202.5 (134-296.7)	435.4 (370.5-566.5)	**$<1x10^{-10}$**	-	-	-	-	-	-	-
ENA-78	202.2 (127.2-280.4)	353.3 (238.3-484.3)	**$<1x10^{-5}$**	-	-	-	-	-	-	-
CD30	1439.1 (945.3-2274.9)	3211.7 (2337.3-4428.7)	**$<1x10^{-5}$**	-	-	-	-	-	-	-
TNF-RII	172.4 (118.1-216.2)	284.8 (233.7-405.4)	**$<1x10^{-10}$**	-	-	-	-	-	-	-
BAFF	139.7 (81.3-177.8)	362.4 (278.5-593.4)	**$<1x10^{-10}$**	-	-	-	-	-	-	-
MDC	426 (281.1-526.7)	677.9 (577.1-835.8)	**$<1x10^{-10}$**	-	-	-	-	-	-	-
APRIL	7304.7 (3887.3-10704)	18394.9 (11938.9-33962.1)	**$<1x10^{-10}$**	-	-	-	-	-	-	-
Tweak	4557.8 (3428.9-6340.9)	9792.2 (7997.2-12516.4)	**$<1x10^{-10}$**	-	-	-	-	-	-	-
sTNFr1	-	-	-	4087.6 (2085.2-6517.3)	7951.3 (4984.3-11821.7)	**$<1x10^{-5}$**	5957.4 (3922.7-9991)	13815.3 (9175-20641.9)	14351.2 (9708.5-23711.7)	**$<1x10^{-20}$**
MPO	-	-	-	292057.8 (152041.4-461508.9)	633615.7 (318953.6-1211865.2)	**$4.9x10^{-5}$**	269062 (175736.1-483624.4)	532337.2 (322949.8-1206784.4)	463795 (234525.1-1349336.9)	**$<1x10^{-10}$**
Ang-2	-	-	-	3172.3 (1493.3-5478.9)	6384.5 (3699.9-11171.1)	**$1.6x10^{-5}$**	3217.1 (1910.4-5127.1)	8315.1 (4536.5-16484.4)	9777.4 (5716-22919.1)	**$<1x10^{-20}$**

	**GAinS**			**VANISH**			**LeoPARDS**			
Analyte	Low cytokine cluster	High cytokine cluster	FDR	Low cytokine cluster	High cytokine cluster	FDR	Low cytokine cluster	Intermediate cytokine cluster	High cytokine cluster	FDR
ICAM	-	-	-	232608.7 (160025.8-370906.2)	321250.4 (209874.2-519756.3)	**0.002**	282668.1 (184974.8-440678.7)	328433.4 (186577.9-582882.8)	402553.3 (242763.6-844714.2)	**0.0005**
CCL5	-	-	-	5969.7 (1702.9-15472.4)	4621.4 (1151.1-15554.8)	0.5	-	-	-	-

Table S4. Comparison of baseline variables and clinical outcomes between clusters from the hierarchical cluster analysis models in GAinS, VANISH and LeoPARDS. Continuous variables have been compared with the Mann-Whitney U test or Kruskal-Wallis test and categorical variables with the chi-squared test or Fisher's exact test (if number of events <10). P-values in bold are those <0.05. No adjustment has been made for multiple comparisons. (APACHE=Acute Physiology and Chronic Health Evaluation, COPD=Chronic Obstructive Pulmonary Disease, NYHA= New York Heart Association, GCS=Glasgow Coma Scale, IV=intravenous, IQR=interquartile range, ICU=Intensive Care Unit). * Organ failure is defined as having a Sequential Organ Failure Assessment (SOFA) score of 3 or more. † In VANISH acute renal failure was defined as having acute kidney injury stage 3.

	GAinS			VANISH			LeoPARDS			
	Low cytokine cluster	High cytokine cluster	p-value	Low cytokine cluster	High cytokine cluster	p-value	Low cytokine cluster	Intermediate cytokine cluster	High cytokine cluster	p-value
n	70	54	-	71	84	-	191	208	85	-
Age median (IQR), y	68 (52-77)	69 (62-76)	0.5	65 (54-77)	64 (52-77)	0.46	68 (58-77)	69 (60.3-76)	66 (52-74)	0.13
Men, No./total (%)	42/70 (60)	38/54 (70)	0.23	49/71 (69)	52/84 (62)	0.36	106/191 (55)	116/208 (56)	46/85 (54)	0.97
Caucasian ethnicity, No./total (%)	67/70 (96)	51/52 (98)	0.64	60/71 (85)	68/84 (81)	0.56	178/191 (93)	193/208 (93)	81/85 (95)	0.73
Recent surgical history, No./total (%)	7/69 (10)	11/54 (20)	0.13	14/71 (20)	11/84 (13)	0.26	53/191 (28)	86/208 (41)	37/85 (44)	**0.01**
APACHE II score, median (IQR)	15 (11-20)	16 (12-21)	0.23	23 (18-28)	24 (20-31)	0.07	24 (21-30)	26 (22-31)	26 (21.5-32.5)	**0.02**
Baseline total SOFA score, median (IQR)	6 (3-7)	6 (4-9)	0.11	-	-	-	10 (7-11)	10 (8-13)	11 (8-14)	**0.0008**
Pre-existing conditions, No./total (%)										
Ischemic heart disease	11/70 (16)	7/54 (13)	0.8	17/71 (24)	8/84 (10)	**0.02**	29/191 (15)	26/208 (13)	16/85 (19)	0.37
Severe COPD	-	-	-	5/71 (7)	3/84 (4)	0.47	13/191 (7)	6/208 (3)	4/85 (5)	0.18
COPD	22/70 (31)	12/54 (22)	0.25	-	-	-	-	-	-	-
Chronic kidney failure	12/70 (17)	5/54 (9)	0.29	2/71 (3)	5/84 (6)	0.45	17/191 (9)	15/208 (7)	3/85 (4)	0.28
Cirrhosis	1/70 (1)	1/54 (2)	1	0/71 (0)	10/84 (12)	**0.002**	5/191 (3)	4/208 (2)	0/85 (0)	0.37
Cancer	9/70 (13)	8/54 (15)	0.8	6/71 (9)	12/84 (14)	0.32	-	-	-	-
Immunocompromised	-	-	-	0/71 (0)	10/84 (12)	**0.002**	14/191 (7)	19/208 (9)	11/85 (13)	0.33
Diabetes	10/70 (14)	7/54 (13)	1	14/71 (20)	17/84 (20)	0.94	41/191 (21)	49/208 (24)	14/85 (16)	0.41
Cardiac Failure	2/70 (3)	0/54 (0)	0.5	-	-	-	16/191 (8)	20/208 (10)	8/85 (9)	0.91
NYHA Heart failure class IV	-	-	-	-	-	-	1/191 (1)	4/208 (2)	0/85 (0)	0.34
*Organ failure, No./total (%)**										
Cardiovascular	25/70 (36)	25/54 (46)	0.23	-	-	-	190/191 (99)	207/208 (100)	84/85 (99)	0.77
Respiratory	10/70 (14)	12/54 (22)	0.25	21/70 (30)	35/83 (42)	0.12	78/189 (41)	69/208 (33)	40/85 (47)	0.06
Kidney †	12/70 (17)	11/54 (20)	0.65	13/71 (18)	22/84 (26)	0.24	13/191 (7)	24/207 (12)	14/84 (17)	**0.04**
Liver	0/70 (0)	2/54 (4)	0.19	4/60 (7)	6/77 (8)	1	3/187 (2)	8/202 (4)	2/85 (2)	0.37
Hematological	0/70 (0)	1/54 (2)	0.44	2/69 (3)	7/82 (9)	0.18	4/190 (2)	12/207 (6)	10/84 (12)	**0.004**
Neurological	3/70 (4)	1/54 (2)	0.63	24/69 (35)	26/80 (33)	0.77	91/167 (54)	85/176 (48)	38/67 (57)	0.37

	GAinS			VANISH			LeoPARDS			
	Low cytokine cluster	High cytokine cluster	p-value	Low cytokine cluster	High cytokine cluster	p-value	Low cytokine cluster	Intermediate cytokine cluster	High cytokine cluster	p-value
Physiological variables, median (IQR)										
Mean arterial pressure, mmHg	64 (55-71)	60 (56-70)	0.67	69 (61-78)	69 (62-75)	0.41	75 (70-81)	73.5 (67-79)	71 (65-75.5)	**0.002**
Highest heart rate, beats/min	110 (97-130)	116 (100-134)	0.35	-	-	-				
Lowest heart rate, beats/min	76 (65-86)	82 (74-88)	**0.008**	85 (75-101)	100 (90-118)	**$<1x10^{-4}$**	86 (73-100)	98 (84-113)	110 (92-121)	**$<1x10^{-13}$**
Central venous pressure, mmHg	-	-	-	13 (7-19)	14 (10-19)	0.35	10 (8-15)	12 (9-16)	12 (9-14.3)	0.33
Lactate, mmol/L	2.2 (1.6-3.4)	2.3 (1.4-3.5)	0.98	1.8 (1.2-2.8)	3.0 (2.0-5.0)	**$<1x10^{-6}$**	1.5 (1-2.2)	2.7 (1.8-3.9)	3.9 (2.5-6.2)	**$<1x10^{-28}$**
PaO_2/FiO_2, kPa	20.6 (13.4-30.1)	16.0 (10.7-24.7)	**0.048**	36.1 (21.3-44.3)	21.3 (14.5-34.6)	**0.0003**	28.3 (21.2-39.7)	30.1 (21.3-40.3)	25.8 (16.4-35.1)	**0.01**
Creatinine, µmol/L	96 (60-141)	95 (73-179)	0.39	94 (67-163)	134 (91-248)	**0.004**	103 (69-166)	155 (110-233)	160 (120-255)	**$<1x10^{-10}$**
Bilirubin, µmol/L	11 (7-18)	11 (8-24)	0.23	11 (7-21)	17 (11-39)	**0.005**	12 (7-18)	17 (9-31)	17 (9-31)	**$<1x10^{-6}$**
Platelets, $\times 10^3/\mu L$	194 (153-273)	190 (154-257)	0.8	206 (151-342)	170 (96-264)	**0.03**	244 (181-351)	208 (131-299)	164 (87-241)	**$<1x10^{-7}$**
GCS	15 (15-15)	15 (15-15)	0.45	13 (5-15)	14 (4-15)	0.64	6 (3-15)	10.5 (3-15)	3 (3-15)	0.31
Bicarbonate, mmol/L	24.0 (20.0-28.6)	22.3 (20.0-24.0)	**0.034**	-	-	-	-	-	-	-
Highest white cell count, $x10^3$ / µL	13.0 (9.9-17.7)	13.8 (9.0-18.4)	0.8	-	-	-	-	-	-	-
Highest temperature, °C	37.3 (36.9-37.8)	37.9 (37.1-38.5)	**0.009**	-	-	-	-	-	-	-
Mechanical ventilation, No./total (%)	47/70 (67)	34/54 (63)	0.63	39/71 (55)	43/84 (51)	0.64	151/191 (79)	166/208 (80)	73/85 (86)	0.39
Renal replacement therapy, No./total (%)	5/70 (7)	6/54 (11)	0.53	2/71 (3)	3/84 (4)	1	15/191 (8)	47/208 (23)	24/85 (28)	**$<1x10^{-4}$**
Volume of IV fluid in previous 4 h, median (IQR), mL	-	-	-	850 (439-1499)	1250 (610-2050)	**0.03**	673 (411-1000)	725 (366-1111)	1087 (599-1568)	**$<1x10^{-4}$**
Noradrenaline dose at randomization, median (IQR), µg/kg/min	-	-	-	0.13 (0.08-0.20)	0.17 (0.10-0.31)	**0.03**	0.21 (0.13-0.36)	0.30 (0.17-0.49)	0.47 (0.26-0.76)	**$<1x10^{-11}$**

	GAinS			VANISH			LeoPARDS			
	Low cytokine cluster	High cytokine cluster	p-value	Low cytokine cluster	High cytokine cluster	p-value	Low cytokine cluster	Intermediate cytokine cluster	High cytokine cluster	p-value
Source of infection, No./total (%)										
Lung	53/70 (76)	39/54 (72)	0.66	31/69 (45)	34/83 (41)	0.62	105/190 (55)	62/208 (30)	22/85 (26)	**<1x10^{-7}**
Abdomen	17/70 (24)	15/54 (28)		12/69 (17)	21/83 (25)	0.24	48/190 (25)	89/208 (43)	40/85 (47)	**0.0001**
Soft tissue or line	-	-		2/69 (3)	3/83 (4)	1	11/190 (6)	7/208 (3)	7/85 (8)	0.19
Other	-	-		24/69 (35)	25/83 (30)	0.54	26/190 (14)	50/208 (24)	16/85 (19)	**0.03**
Vasopressor or inotrope administration, No./total (%)	25/70 (36)	26/54 (48)	0.23	-	-	-	-	-	-	-
Inotropes No./total (%)	-	-	-	2/71 (3)	20/84 (24)	**0.0001**	-	-	-	-
SRS1 No./total (%)	35/66 (53)	29/49 (59)	0.51	18/68 (26)	51/81 (63)	**<1x10^{-5}**	-	-	-	-
SRS2 No./total (%)	31/66 (47)	20/49 (41)		50/68 (74)	30/81 (37)		-	-	-	-
**Outcomes**										
28-d Mortality, No./total (%)	13/69 (19)	14/54 (26)	0.35	14/71 (20)	30/84 (36)	**0.03**	46/190 (24)	71/208 (34)	38/85 (45)	**0.002**
ICU mortality, No./total (%)	10/69 (14)	14/54 (26)	0.11	12/71 (17)	24/84 (29)	0.09	48/191 (25)	61/208 (29)	38/85 (45)	**0.004**
Hospital mortality, No./total (%)	19/69 (27)	17/54 (26)	0.63	14/71 (20)	28/84 (33)	0.06	54/190 (28)	75/208 (36)	39/85 (46)	**0.02**
3-month Mortality, No./total (%)	21/69 (30)	17/54 (31)	0.9	-	-	-	57/188 (30)	78/208 (38)	42/85 (49)	**0.01**
6-month Mortality, No./total (%)	23/69 (33)	19/54 (35)	0.82	-	-	-	60/188 (32)	86/208 (41)	43/85 (51)	**0.01**
Kidney failure, No./total (%)	16/70 (23)	14/54 (26)	0.69	28/71 (39)	43/84 (51)	0.14	-	-	-	-
Kidney failure free days, median (IQR), days	-	-	-	22 (5-26)	8 (0-22)	**0.02**	-	-	-	-
Duration of renal replacement therapy, median (IQR), days	-	-	-	-	-	-	0 (0-0)	0 (0-4)	1 (0-5)	**<1x10^{-8}**

	**GAinS**			**VANISH**			**LeoPARDS**			
	Low cytokine cluster	High cytokine cluster	p-value	Low cytokine cluster	High cytokine cluster	p-value	Low cytokine cluster	Intermediate cytokine cluster	High cytokine cluster	p-value
No. weaned from vasopressors for >24 h, No./total (%)	-	-	-	68/71 (96)	73/84 (87)	0.06	-	-	-	-
Time to shock reversal, median (IQR), hours	-	-	-	43 (24-84)	36 (16-73)	0.24	-	-	-	-
Duration of inotrope/vasopressor support, median(IQR), days	0 (0-3)	1.5 (0-5)	**0.026**	-	-	-	-	-	-	-
Catecholamine free days, median (IQR), days	-	-	-	-	-	-	24 (4.5-26)	23 (0-26)	14 (0-25)	**0.0002**
Duration of mechanical ventilation, median (IQR), days	3 (1-8)	4 (1-12)	0.2	6 (3-11)	5 (2-11)	0.27	-	-	-	-
Ventilator free days, median (IQR), days	-	-	-	-	-	-	20 (0.5-27)	19 (0-26)	2 (0-20)	**$<1x10^{-5}$**
Mean total SOFA score over ICU stay, median (IQR)	-	-	-	3.9 (3.0-5.5)	5.7 (4.0-9.4)	**$<1x10^{-4}$**	4.1 (2.9-6.1)	5.9 (3.9-9.0)	7.3 (4.3-13.0)	**$<1x10^{-12}$**
ICU length of stay, median (IQR), days	6 (4-11)	8 (4-14)	0.3	6 (3-12)	6 (3-12)	0.72	7.9 (4-14.6)	8 (3.7-13.6)	7.9 (2.4-14.8)	0.48
Hospital length of stay, median (IQR), days	19.0 (9.0-31.0)	16.5 (10.0-32.0)	0.95	21 (10-43)	15 (6-33)	**0.03**	22.6 (11.8-40.3)	22.4 (12.2-43.4)	17.7 (4.7-51.3)	0.28

Table S5 Fit statistics for latent class models. Mean class probability is averaged over all class members. For LeoPARDS, we fit the full model with all class-predicting covariates and non-zero covariance between continuous indicators. For VANISH a reduced set of covariates was used to achieve convergence, and zero covariance between indicators was assumed based on model fit (see results for details). AIC= Akaike Information Criterion, BIC= Bayesian Information Criterion.

						AIC	BIC	Entropy
LeoPARDS		Class 1	Class 2	Class 3	Class 4			
1 class	# per class	493				25091	25404	-
	Mean class probability	1.00						
2 classes	# per class	283	210			23833	24339	0.87
	Mean class probability	0.97	0.96					
3 classes	# per class	191	247	55		23238	23936	0.91
	Mean class probability	0.94	0.96	0.98				
4 classes	# per class	151	132	156	54	22997	23887	0.88
	Mean class probability	0.95	0.93	0.90	0.98			
VANISH								
1 class	# per class	176				7714	7874	
	Mean class probability	1.00						
2 classes	# per class	90	86			7378	7671	0.88
	Mean class probability	0.96	0.97					
3 classes	# per class	46	67	63		7298	7722	0.86
	Mean class probability	0.98	0.90	0.95				
4 classes	# per class	30	41	43	62	7281	7838	0.88
	Mean class probability	0.95	0.93	0.91	0.95			

Table S6 Estimated class distribution, indicator means and separation for the LeoPARDS trial. In these models, all covariates were included and the residual variance of each indicator can differ across classes.

	**2-class model**			**3-class model**				**4-class model**				
	**Class 1**	**Class 2**	**Separation**	**Class 1**	**Class 2**	**Class 3**	**Separation**	**Class 1**	**Class 2**	**Class 3**	**Class 4**	**Separation**
Distribution (%)	58	42		39	50	11		31	25	33	11	
Organ dysfunction												
PaO_2/FiO_2 ratio	0.12	-0.15	0.018	0.098	-0.01	-0.252	0.021	0.076	0.109	-0.096	-0.232	0.019
Creatinine	-0.228	0.289	0.067	-0.368	0.155	0.451	0.115	-0.512	0.233	0.078	0.459	0.129
Platelets	0.238	-0.319	0.078	0.304	-0.087	-0.58	0.131	0.378	-0.335	0.119	-0.627	0.152
Bilirubin	-0.231	0.284	0.066	-0.295	0.196	0.021	0.041	-0.454	0.585	-0.01	0.054	0.136
Inflammation markers												
IL-1β	-0.587	0.416	0.252	-0.774	-0.036	1.383	0.801	-0.825	-0.5	0.157	1.381	0.713
IL-6	-0.487	1.123	0.648	-0.746	0.428	2.506	1.808	-0.773	-0.369	0.795	2.456	1.571
IL-8	-0.552	0.838	0.483	-0.836	0.274	1.954	1.315	-0.925	-0.173	0.45	1.953	1.119
IL-10	-0.532	0.775	0.427	-0.779	0.283	1.601	0.948	-0.894	0.058	0.344	1.62	0.807
IL-17	-0.4	0.465	0.187	-0.499	0.106	0.9	0.328	-0.565	0.079	0.079	0.924	0.28
IL-18	-0.258	0.479	0.136	-0.358	0.246	0.569	0.148	-0.497	0.641	-0.053	0.605	0.226
Leukocytes												
Myeloperoxidase	-0.373	0.303	0.114	-0.531	0.125	0.461	0.17	-0.64	0.194	-0.008	0.49	0.172
sICAM	-0.2	0.515	0.128	-0.175	0.166	0.742	0.143	-0.312	0.823	-0.195	0.777	0.279
Endothelial injury												
Angiotensin-2	-0.486	0.696	0.349	-0.709	0.356	0.894	0.444	-0.896	0.556	0.152	0.933	0.467
Cardiovascular												
Troponin	-0.017	0.018	0	-0.034	-0.017	0.157	0.007	-0.09	0.126	-0.128	0.18	0.018
B-natriuretic peptide	-0.368	0.07	0.048	-0.346	-0.184	0.361	0.091	-0.496	0.166	-0.359	0.359	0.126
Other markers												
sTNFr-1	-0.414	0.56	0.237	-0.659	0.333	0.705	0.331	-0.865	0.404	0.229	0.719	0.355
Lactate	-0.425	0.59	0.258	-0.613	0.263	0.91	0.39	-0.684	0.048	0.342	0.898	0.326
MCP-1 (CCL2)	-0.533	0.873	0.494	-0.753	0.262	1.944	1.237	-0.817	-0.087	0.369	1.966	1.041

Table S7: Differences in class assignment for main and sensitivity analysis in latent class analysis

**Main analysis**	**Sensitivity analysis**		
LeoPARDS	Class 1	Class 2	Class 3
Class 1	180	11	0
Class 2	5	228	14
Class 3	0	0	55
VANISH	Class 1	Class 2	
Class 1	87	3	
Class 2	2	84	

Table S8: Confusion matrices comparing hierarchical cluster analysis cluster assignment and latent class for A) LeoPARDS and B) VANISH

A

		Latent class analysis group		
**Hierarchical cluster analysis group**		Class 1	Class 2	Class 3
	High cytokine cluster	0	34	51
	Intermediate cytokine cluster	22	185	1
	Low cytokine cluster	167	24	0

B

		Latent class analysis group	
**Hierarchical cluster analysis group**		Class 1	Class 2
	High cytokine cluster	12	72
	Low cytokine cluster	68	3

Table S9: Latent class analysis variables by class, with study participants assigned by highest posterior class probability in latent class analysis. Variable values are median (interquartile range) for values within the limit of detection. The number of values outside the limit of detection as a percentage of non-missing values is given.

	LeoPARDS (N=493)				VANISH (N=176)		
	Class-1 N=191	Class-2 N=247	Class-3 N=55	n (%) outside limits	Class-1 N=90	Class-2 N=86	n (%) outside limits
Organ dysfunction							
PaO_2/FiO_2 ratio (kPa)	29.1 (22, 39.7)	29.3 (20.2, 39.4)	25.8 (16.6, 36)	0/491	32.5 (21, 43.5)	21.1 (14.6, 34.9)	0/169
Creatinine (µmol/l)	107 (69, 166)	151 (107, 231)	173 (137, 295)	0/491	91.5 (67, 163)	140 (106, 270)	0/176
Platelets ($x10^9/l$)	243 (182, 350)	203 (131, 294)	136 (76, 215)	0/490	206 (145, 335)	150 (83, 246)	0/171
Bilirubin (µmol/l)	12 (7, 19)	17 (10, 30)	17 (9, 31)	0/483	12 (7, 23)	16.5 (11, 42)	0/156
Inflammation markers							
IL-1β (pg/ml)	0.915 (0.651, 1.41)	1.53 (0.948, 2.88)	7.96 (2.93, 11.3)	43/486 (8.8)	4.9 (4.34, 6.25)	11.2 (7.71, 19.4)	109/162 (67.3)
IL-6 (pg/ml)	232 (92, 481)	1588 (583, 3874)	19582 (11926, 27584)	34/490 (6.9)	426 (175, 1376)	6385 (2277, 19641)	15/162 (9.3)
IL-8 (pg/ml)	48.2 (30.4, 84.8)	257 (159, 516)	3015 (1252, 7336)	4/490 (0.8)	64.8 (28.3, 173)	1075 (225, 3293)	16/162 (9.9)
IL-10 (pg/ml)	26.3 (17.4, 49.8)	123 (66.1, 205)	554 (314, 1429)	1/490 (0.2)	15.1 (8.27, 32.9)	159 (66, 446)	16/162 (9.9)
IL-17 (pg/ml)	6.61 (4.71, 10.1)	9.79 (6.52, 19.5)	21.5 (8.1, 49.9)	9/486 (1.9)	15.6 (7.36, 22.6)	18 (10.6, 38.8)	108/162 (66.7)
IL-18 (pg/ml)	559 (373, 996)	804 (565, 1278)	1065 (724, 1759)	16/486 (3.3)	434 (163, 595)	562 (331, 836)	3/162 (1.9)
Leukocytes							
Myeloperoxidase (pg/ml)	332192 (204356, 581390)	489569 (291316, 987773)	541091 (340269, 1405943)	47/486 (9.7)	264802 (122264, 433825)	675769 (448047, 1195365)	3/168 (1.8)
sICAM (pg/ml)	271181 (168034, 414706)	311864 (187557, 515209)	432439 (298658, 886124)	23/486 (4.7)	243841 (165654, 326461)	341912 (208505, 550529)	6/168 (3.6)
Endothelial injury							
Angiotensin-2 (pg/ml)	3197 (1906, 5419)	7487 (4491, 14867)	13040 (7373, 23168)	15/486 (3.1)	3162 (1503, 5551)	6592 (3681, 11564)	3/168 (1.8)
Cardiovascular							
Troponin (ng/L)	62 (16.8, 536)	77.8 (23.7, 381)	139 (45.2, 589)	0/483	21 (6, 94)	149 (31, 729)	0/95
B-natriuretic peptide (pg/ml)	9054 (3318, 17410)	10269 (4922, 23317)	18406 (9844, 31718)	36/492 (7.3)	3611 (1238, 7416)	8130 (3590, 16890)	3/168 (1.8)
Other markers							

sTNFr-1 (pg/ml)	5939 (3923, 9802)	13457 (8749, 20337)	18099 (12379, 27759)	0/492	3856 (2064, 5555)	8315 (5743, 12645)	2/168 (1.2)
Lactate (mmol/l)	1.5 (1, 2.1)	2.6 (1.8, 4)	5.2 (3, 7)	0/490	1.8 (1.2, 2.5)	3.5 (2.3, 5.3)	0/172
MCP-1 (CCL2) (pg/ml)	384 (272, 592)	995 (676, 1590)	4049 (3105, 5621)	6/490 (1.2)	-	-	-

Table S10: Baseline characteristics and outcomes overall and by latent class analysis assigned class. Values are presented as median (interquartile range) for continuous variables and n (%) for categorical variables.

	LeoPARDS N=493					VANISH N=176			
Class	Overall N=493	Class-1 N=191	Class-2 N=247	Class-3 N=55	missing (n)	Overall N=176	Class-1 N=90	Class-2 N=86	missing (n)
Age (years)	68 (58, 76)	68 (57, 77)	69 (62, 76)	65 (51, 73)	0	65 (53.5, 77)	65.5 (54, 77)	64.5 (53, 76)	0
Male	274 (56)	108 (56.5)	138 (55.9)	28 (50.9)	0	112 (63)	58 (64.4)	54 (62.8)	0
Ethnicity					0				0
Caucasian	461 (94)	180 (94.2)	229 (92.7)	52 (94.6)		146 (83)	77 (85.6)	69 (80.2)	
Black	10 (2)	3 (1.6)	5 (2)	2 (3.6)		14 (8)	8 (8.9)	6 (7)	
Asian	19 (4)	7 (3.7)	11 (4.5)	1 (1.8)		13 (7)	4 (4.4)	9 (10.5)	
Other	3 (1)	1 (0.5)	2 (0.8)	0 (0)		3 (2)	1 (1.1)	2 (2.3)	
BMI	27.1 (23.4, 31.0)	26.7 (23, 30.8)	27.3 (23.4, 30.7)	27.8 (24.2, 33.6)	6	26.1 (22.5, 31.3)	24.7 (22.2, 31.6)	26.2 (22.6, 31.1)	9
Co-morbidities									
NYHA Class IV	5 (1)	1 (0.5)	4 (1.6)	0 (0)	0	0 (0)	0 (0)	0 (0)	0
Severe COPD	23 (5)	10 (5.2)	11 (4.5)	2 (3.6)	0	10 (6)	7 (7.8)	3 (3.5)	0
Chronic renal failure	35 (7)	18 (9.4)	15 (6.1)	2 (3.6)	0	8 (5)	2 (2.2)	6 (7)	0
Cirrhosis	9 (2)	6 (3.1)	3 (1.2)	0 (0)	0	11 (6)	4 (4.4)	7 (8.1)	0
Immunocompromised	45 (9)	14 (7.3)	22 (8.9)	9 (16.4)	0	11 (6)	2 (2.2)	9 (10.5)	0
Site of infection					3				1
Lung	192 (39)	106 (55.5)	74 (30.1)	12 (21.8)		74 (42)	42 (48.3)	32 (37.2)	
Abdomen	181 (37)	45 (23.6)	111 (45.1)	25 (45.5)		35 (20)	17 (19.5)	18 (20.9)	
Urine	29 (6)	13 (6.8)	13 (5.3)	3 (5.5)		28 (16)	10 (11.5)	18 (20.9)	
Primary bacteremia	10 (2)	0 (0)	6 (2.4)	4 (7.3)		3 (2)	2 (2.3)	1 (1.2)	
Neurological	5 (1)	3 (1.6)	2 (0.8)	0 (0)		4 (2)	4 (4.6)	0 (0)	
Soft tissue or line	26 (5)	10 (5.2)	10 (4.1)	6 (10.9)		6 (3)	3 (3.5)	3 (3.5)	
Other	49 (10)	14 (7.3)	30 (12.2)	5 (9.1)		23 (13)	9 (10.3)	14 (16.3)	
SOFA	8 (6, 9)	7 (6,8)	8 (7,10)	9 (7,11)	22	7 (5, 9)	6 (4,8)	8 (5,10)	16
APACHE II	25 (21, 31)	24 (21, 30)	26 (21, 31)	27 (22, 30)	1	24 (19, 30)	24 (18, 29)	23.5 (20, 30)	0
Post-surgical admission	180 (37)	49 (25.7)	114 (46.2)	17 (30.9)	0	26 (15)	17 (18.9)	9 (10.5)	0
Class	Overall N=493	Class-1 N=191	Class	Overall N=493	Class-1 N=191	Class	Overall N=493	Class-1 N=191	Class

	LeoPARDS N=493					VANISH N=176			
**Outcomes**					p-value for difference				p-value for difference
Mean daily SOFA, mean (SD)		4.93 (2.88)	6.67 (3.94)	9.97 (4.60)	<0.001†				
3-month survival, n/N (%)		132/189 (69.8%)	155/246 (63.0%)	23/55 (41.8%)	0.001*				
28-day survival, n/N (%)		143/190 (75.3%)	165/247 (66.8%)	26/55 (47.3%)	<0.001*		72/90 (80.0%)	57/86 (66.3%)	0.04*
28-day renal failure-free survival, n/N (%)[1]							51/75 (68.0%)	32/60 (53.3%)	0.08*
Renal failure-free days, median (IQR)[2]							19 (3,26)	8 (0, 23)	0.03‡

NYHA= New York Heart Association, COPD=Chronic Obstructive Pulmonary Disease, SOFA=Sequential Organ Failure Assessment, APACHE=Acute Physiology and Chronic Health Evaluation; * χ^2 test; † Kruskal-Wallis test; ‡ Mann-Whitney test; [1] in patients not in renal failure at baseline; [2] in patients who die or experience some renal failure by day-28.

Table S11: Confusion matrices comparing ARDS parsimonious model allocations (model 1: IL-8, sTNFr1, vasopressors and model 2: IL-6, sTNFr1, vasopressors) with hierarchical cluster and latent class analysis group assignments for A) LeoPARDS and B) VANISH. Class assignment from the ARDS models were established using a probability cut-off of ≥0.5 for membership of the hyper-inflammatory group. The percentages labelled represent those out of the total number in the corresponding latent class or hierarchical cluster.

A) LeoPARDS

		**Latent class analysis group**			
**ARDS model 1**		Class 1	Class 2	Class 3	Total
	Hyper-inflammatory	132 (69%)	244 (99%)	54 (100%)	430 (88%)
	Hypo-inflammatory	58 (31%)	2 (1%)	0 (0%)	60 (12%)
	Total	190	246	54	490
**ARDS model 2**	Hyper-inflammatory	122 (64%)	244 (99%)	54 (100%)	420 (86%)
	Hypo-inflammatory	68 (36%)	2 (1%)	0 (0%)	70 (14%)
	Total	190	246	54	490
		**Hierarchical clustering group**			
**ARDS model 1**		Low Cytokine	Intermediate Cytokine	High Cytokine	Total
	Hyper-inflammatory	132 (69%)	207 (100%)	85 (100%)	424 (88%)
	Hypo-inflammatory	59 (31%)	1 (0%)	0 (0%)	60 (12%)
	Total	191	208	85	484
**ARDS model 2**	Hyper-inflammatory	122 (64%)	207 (100%)	85 (100%)	414 (86%)
	Hypo-inflammatory	69 (36%)	1 (0%)	0 (0%)	70 (14%)
	Total	191	208	85	484

B) VANISH

		**Latent class analysis group**		
**ARDS model 1**		Class 1	Class 2	Total
	Hyper-inflammatory	39 (48%)	74 (97%)	113 (72%)
	Hypo-inflammatory	42 (52%)	2 (3%)	44 (28%)
	Total	81	76	157
**ARDS model 2**	Hyper-inflammatory	49 (43%)	76 (100%)	125 (80%)
	Hypo-inflammatory	32 (28%)	0 (0%)	32 (20%)
	Total	113	76	157
		**Hierarchical clustering group**		
**ARDS model 1**		Low Cytokine	High Cytokine	Total
	Hyper-inflammatory	31 (44%)	80 (95%)	111 (72%)
	Hypo-inflammatory	40 (56%)	4 (5%)	44 (28%)
	Total	71	84	155
**ARDS model 2**	Hyper-inflammatory	40 (56%)	84 (100%)	124 (80%)
	Hypo-inflammatory	31 (44%)	0 (0%)	31 (20%)
	Total	71	84	155

Table S12: The assessment of heterogeneity of treatment effect in the VANISH and LeoPARDS trials by cytokine hierarchical cluster. Categorical variables are given as n/N (percentage) and continuous variables are given as median (interquartile range), p-values represent the p-value for interaction between the cluster assignment and drug allocation on the outcome.

	**High Cytokine Cluster**		**Intermediate Cytokine Cluster**		**Low Cytokine Cluster**		
**VANISH**							
**Outcome**	**Noradrenaline**	**Vasopressin**			**Noradrenaline**	**Vasopressin**	**p-value**
Day-28 survival	26/42 (62)	28/42 (67)			31/35 (89)	26/36 (72)	0.10*
Renal failure free survival	16/31 (52)	18/31 (58)			21/27 (78)	18/31 (58)	0.13*
renal failure free days	2 (0-21)	13.5 (1-23.5)			25 (15-26)	14.5 (1-26)	0.17†
	**Placebo**	**Hydrocortisone**			**Placebo**	**Hydrocortisone**	
Day-28 survival	20/32 (63)	19/30 (63)			18/21 (86)	12/19 (63)	0.17*
Renal failure free survival	11/24 (46)	13/21 (62)			13/18 (72)	8/15 (53)	0.12*
Renal failure free days	14 (0-23)	7 (0-18)			22.5 (17.5-26)	9 (0-26)	0.28†
**LeoPARDS**							
	**Placebo**	**Levosimendan**	**Placebo**	**Levosimendan**	**Placebo**	**Levosimendan**	
3-month survival	26/47 (55)	17/38 (45)	64/104 (62)	66/104 (63)	66/88 (75)	65/100 (65)	0.37*
Day-28 survival	29/47 (62)	18/38 (47)	68/104 (65)	69/104 (66)	71/89 (80)	73/101 (72)	0.41*
Mean Total SOFA	6.1 (4.3-13.0)	9.3 (5.1-13.3)	5.7 (3.9-8.2)	6.1 (3.9-9.0)	3.7 (2.6-5.3)	4.6 (3.4-6.5)	0.74, 0.73‡

SOFA = Sequential Organ Failure Assessment, *binary logistic regression, † aligned rank transform test, ‡logistic regression of square root transformed outcome variables where p-values given are for interaction term for the intermediate and low cytokine clusters with the high cluster as the reference

Table S13: Differential plasma cytokine and related protein abundance analysis between the SRS1 and SRS2 transcriptomic sub-phenotypes in GAinS and VANISH. Concentrations in each group are given as median and interquartile range in units of pg/mL. Comparisons are made with the Mann-Whitney U test. FDR values in bold are those <0.05. A fold change larger than one indicates higher median level in SRS1.

Plasma protein	GAinS				VANISH			
	SRS1	SRS2	Fold Change	FDR	SRS1	SRS2	Fold Change	FDR
n	64	51	-	-	69	80	-	-
MCP-1	400.2 (218.8-739.1)	223.2 (118.8-369.3)	1.79	**0.005**	2998.9 (1283.9-7679.1)	1289.8 (595.2-3636.6)	2.33	**<0.001**
IL-6	1768.5 (1185-2843.8)	1043 (716.3-1436.7)	1.7	**0.0008**	8253.6 (1557.1-32205.6)	497.1 (177.1-2931)	16.6	**<1x10^{-7}**
IL-8	516.4 (376.4-633.1)	386.7 (195.4-556.8)	1.34	**0.03**	288.2 (54.8-2446.2)	93.5 (26-588.3)	3.08	**0.01**
IL-10	245.9 (147.5-418.9)	195.9 (99.6-354.1)	1.25	0.34	107.2 (27.7-375.4)	17.8 (6-57.1)	6.01	**<1x10^{-5}**
IL-18	875.9 (599.1-1279.7)	872.6 (557.1-1136.2)	1	0.62	462.1 (236.9-665.8)	471.4 (217.7-881.8)	0.98	0.52
CCL3	106.8 (80.1-156.3)	83 (61.9-121.6)	1.29	**0.03**	54.1 (15.4-135.3)	20.2 (8.6-53.5)	2.67	**<0.01**
IP-10	544.6 (341.1-787.5)	359.5 (243.5-477.7)	1.52	**0.02**	834 (344-3496.7)	581.5 (264.6-1322.3)	1.43	0.14
IFN-γ	668 (504.9-976.3)	572.3 (423.9-908.5)	1.17	0.31	4.9 (2.4-45.2)	6.3 (2.4-29.8)	0.78	0.81
IL-1β	61.4 (28.3-143.2)	58.8 (19-136.8)	1.05	0.48	3.6 (2.8-5.9)	3.6 (2.8-5.5)	1	0.84
IL-2	33.4 (23.7-42.8)	25.6 (22-42.3)	1.3	0.27	5.1 (3.3-12.2)	6.4 (4.1-13.6)	0.8	0.78
IL-17	713.5 (546.4-1133.8)	602.4 (366.6-943.9)	1.18	0.1	4.6 (3.1-12.8)	6.3 (4.6-15.3)	0.73	0.15
TNF-α	84.1 (54.5-131)	82.9 (49.7-138.2)	1.01	0.62	3.1 (2.4-5.8)	4 (3-6.1)	0.78	0.41
IFN-α	123 (75-155.7)	89.9 (75-167)	1.37	0.4	3.7 (2.7-6.4)	3.7 (3-5.4)	1	0.81
IL-12p70	84.8 (46.1-144.2)	96.1 (35.3-169.3)	0.88	0.74	2.9 (2.4-4.7)	3.7 (2.9-4.5)	0.79	0.52
IL-4	2540.6 (1683-3159.6)	2569.2 (1551.7-3640.8)	0.99	0.85	4.9 (2.6-5.7)	4.9 (2.2-5.7)	1	0.44
IL-1α	69 (46.6-154.6)	65.8 (29.9-138.5)	1.05	0.46	4.8 (3.1-15.6)	5.3 (3.4-15.6)	0.9	0.94
MCP-2	123 (78.8-200.9)	103.9 (56.9-173.8)	1.18	0.26	-	-	-	-

Plasma protein	GAinS				VANISH			
	SRS1	SRS2	Fold Change	FDR	SRS1	SRS2	Fold Change	FDR
IL-2R	28647.8 (12275.6-42128.9)	15755 (7777.3-24241.3)	1.82	**0.02**	-	-	-	-
SDF-1α	2720 (1664.9-4602.5)	3066 (1482.9-5347.9)	0.89	1	-	-	-	-
IL-27	1581.3 (1087.1-2482.5)	1658 (957.1-2562.5)	0.95	0.74	-	-	-	-
LIF	20.9 (11.7-31.4)	15.8 (6.1-27.9)	1.32	0.1	-	-	-	-
IL-5	123.2 (62.7-250.2)	103.5 (46.3-197.1)	1.19	0.34	-	-	-	-
IL-7	36.4 (21.3-54.8)	30.3 (11.8-50.3)	1.2	0.25	-	-	-	-
BLC	422.8 (236.1-1294.1)	324.2 (182.2-589.1)	1.3	0.1	-	-	-	-
Eotaxin-2	262.1 (160.1-389.3)	274.1 (157.3-462.3)	0.96	0.74	-	-	-	-
Eotaxin	115.2 (90-153)	103.5 (74.2-120.8)	1.11	0.13	-	-	-	-
IL-13	142.2 (107.1-182)	114.3 (71.8-201.1)	1.25	0.39	-	-	-	-
IL-31	1056.3 (668.1-1818.2)	657.9 (318.4-1214.2)	1.61	**0.04**	-	-	-	-
SCF	88.3 (58.7-123.1)	67.1 (43.2-119.3)	1.32	0.26	-	-	-	-
G-CSF	202.3 (75.7-398.3)	113.3 (39.5-264.8)	1.79	0.13	-	-	-	-
GM-CSF	243.4 (163.4-471.3)	286.3 (140.3-521.8)	0.85	0.74	-	-	-	-
HGF	2521.9 (1512.6-5254.2)	1584.4 (1089.4-2279)	1.59	**0.007**	-	-	-	-
MIP-1β	285.6 (184.5-452.2)	245.9 (130.1-342.9)	1.16	0.2	-	-	-	-
Eotaxin-3	129 (84.1-167.3)	113.3 (84.8-170)	1.14	0.49	-	-	-	-
IL-9	285.6 (193.9-367.1)	212.9 (123.4-369.7)	1.34	0.2	-	-	-	-
MIF	29.6 (18.9-44.7)	22.1 (14.8-39.3)	1.34	0.37	-	-	-	-
TNF-β	639.1 (395.9-920.8)	599.7 (243.1-929)	1.07	0.41	-	-	-	-
bNGF	1018.7 (808.8-1174.3)	909.1 (680.7-1243.4)	1.12	0.62	-	-	-	-
MIP-3α	1280.7 (940.6-2243.3)	1052.4 (510.3-1482.6)	1.22	**0.03**	-	-	-	-

Plasma protein	GAinS				VANISH			
	SRS1	SRS2	Fold Change	FDR	SRS1	SRS2	Fold Change	FDR
I-TAC	521.6 (336.5-858.4)	496.5 (235.8-821.2)	1.05	0.32	-	-	-	-
TRAIL	187.3 (122.8-283.1)	184.9 (112.5-261.9)	1.01	0.74	-	-	-	-
Fractalkine	18.2 (12.7-40.7)	17.3 (8.9-30.2)	1.05	0.4	-	-	-	-
GRO-α	131.8 (77.1-187.3)	91.5 (29.9-128.9)	1.44	0.1	-	-	-	-
IL-23	2345.9 (1536-3165.4)	2395.8 (1390.6-3269.9)	0.98	1	-	-	-	-
MMP-1	107.8 (67.8-174.6)	56.6 (29.3-140.8)	1.91	**0.03**	-	-	-	-
IL-15	32 (13.4-63.8)	19.6 (13.4-62.5)	1.64	0.49	-	-	-	-
M-CSF	1705.7 (1215.7-2730.3)	1669.5 (941.6-2669.6)	1.02	0.66	-	-	-	-
MCP-3	285.7 (184.1-494.7)	245.4 (154.5-388.9)	1.16	0.32	-	-	-	-
MIG	194.2 (122.4-460)	150.1 (72.4-327.4)	1.29	0.16	-	-	-	-
IL-16	758.9 (495.8-1049.5)	672.5 (394.6-1057.2)	1.13	0.66	-	-	-	-
IL-21	245.8 (158.1-340.3)	203.5 (80.3-297.4)	1.21	0.25	-	-	-	-
IL-3	147.5 (94.4-373.8)	139.2 (73.2-344.1)	1.06	0.46	-	-	-	-
CD40-ligand	299.5 (167.5-531.6)	270.9 (147.3-433)	1.11	0.45	-	-	-	-
FGF-2	486.8 (299.1-677.4)	430.1 (259.5-586.2)	1.13	0.4	-	-	-	-
IL-22	583 (419.3-1132.6)	498.9 (286.1-1052.4)	1.17	0.39	-	-	-	-
VEGF-A	517.3 (383.8-825.5)	505.7 (245.5-826.8)	1.02	0.48	-	-	-	-
TSLP	187.4 (134.9-296.6)	187.4 (104.9-250.4)	1	0.39	-	-	-	-
IL-20	293.4 (184-474.3)	313.6 (187.1-404.9)	0.94	0.74	-	-	-	-
ENA-78	285.5 (202.7-447.5)	225.6 (155.4-337)	1.27	0.13	-	-	-	-
CD30	2200.4 (1388.8-4040.6)	2236.4 (1248.3-3447)	0.98	0.9	-	-	-	-
TNF-RII	220.9 (168.9-295.5)	207.2 (129.3-269.9)	1.07	0.32	-	-	-	-
BAFF	226.1 (146.3-384.5)	193.5 (105.3-352.2)	1.17	0.46	-	-	-	-
MDC	508.7 (367.4-658.5)	534.7 (355.8-728.8)	0.95	0.97	-	-	-	-

Plasma protein	GAinS				VANISH			
	SRS1	SRS2	Fold Change	FDR	SRS1	SRS2	Fold Change	FDR
APRIL	10810 (7397.2-23132.6)	9838 (4080.1-14628.1)	1.1	0.16	-	-	-	-
Tweak	6342 (4444-9932.6)	6067 (4162.9-9199.5)	1.05	0.48	-	-	-	-
ICAM	-	-	-	-	286696.2 (209540.6-480708.6)	257379.4 (168244-420958.9)	1.11	0.48
CCL5	-	-	-	-	4650.5 (1661.5-15834.5)	5030.1 (1176-14705.3)	0.92	0.96
sTNFR	-	-	-	-	6356.4 (4039.8-10275.2)	5558.4 (3542.8-9253.7)	1.14	0.48
MPO	-	-	-	-	572165.9 (197021.2-1121886.2)	345249.9 (186685.2-632967.7)	1.66	0.09
Ang-2	-	-	-	-	5478.9 (2858.6-10293.6)	3912.7 (1779.8-7159.3)	1.4	**0.03**

Figure S1: Assessment of hierarchical clustering performance in the full plasma protein of GAinS (left), VANISH (middle) and LeoPARDS (right). (**A**) Test MSE (mean squared error) calculated by performing k-means clustering on 90% of samples and then taking the average of the squared distance of the 10% test samples to the closest cluster centres. 60 train/test sample sets were randomly drawn for each cluster number increasing from two to ten. (**B**) Heatmaps showing the level of consensus index which is the proportion of two samples which were in the same cluster during 1000 iterations following randomly resampling 80% of samples and 95% of plasma proteins each time.

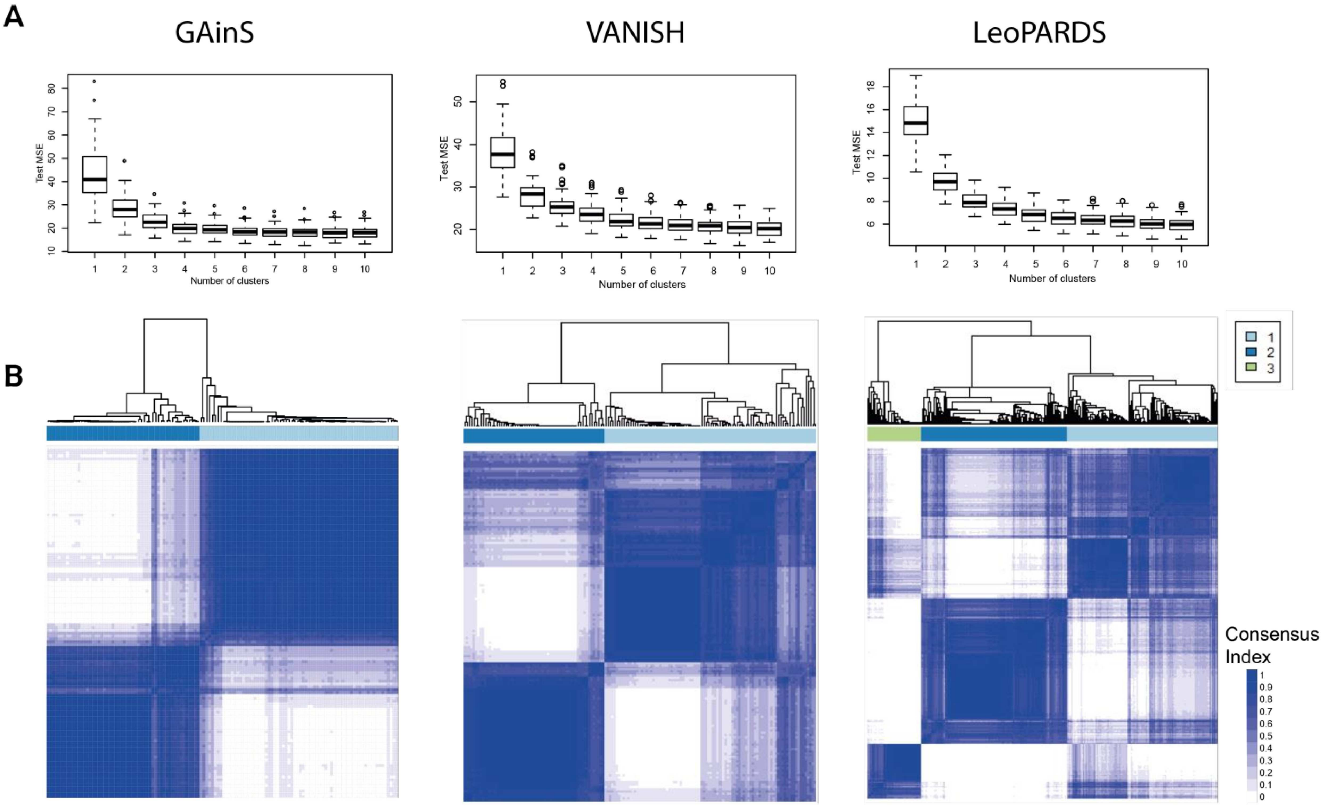

Figure S2: Hierarchical clustering of natural log transformed plasma cytokines and related proteins in baseline samples in A) GAinS, B) VANISH and C) LeoPARDS. Heatmap coloured by protein concentration, patients shown as columns and proteins as rows, solid bars represent Sepsis Response Signature (SRS) assignment (light purple = SRS1, dark purple = SRS2, blank = no assignment available).

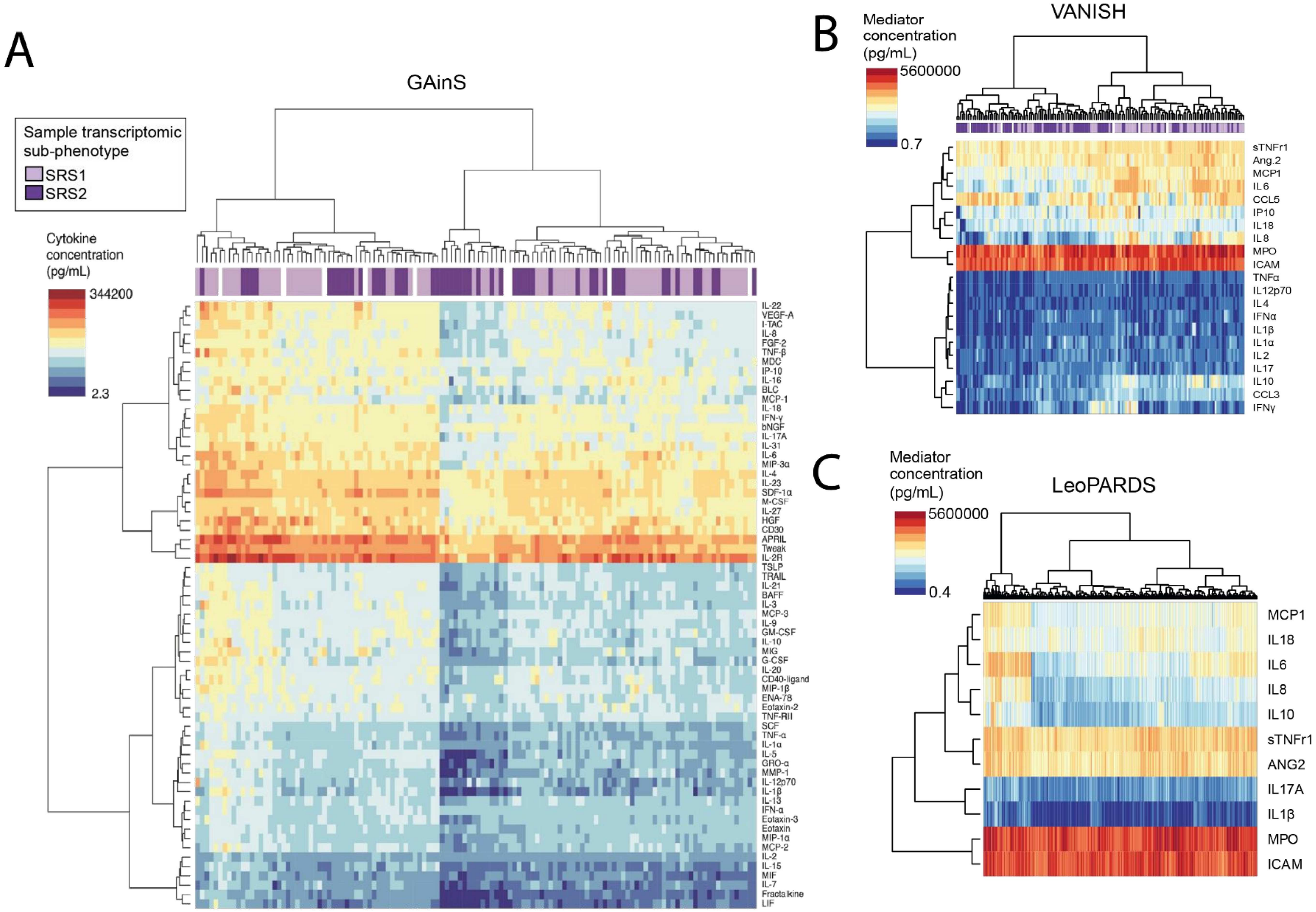

Figure S3: Plots of latent class analysis model fit indicators in the LeoPARDS trial: a) Akaike Information Criterion (AIC); b) Bayesian Information Criterion (BIC) and c) log likelihood

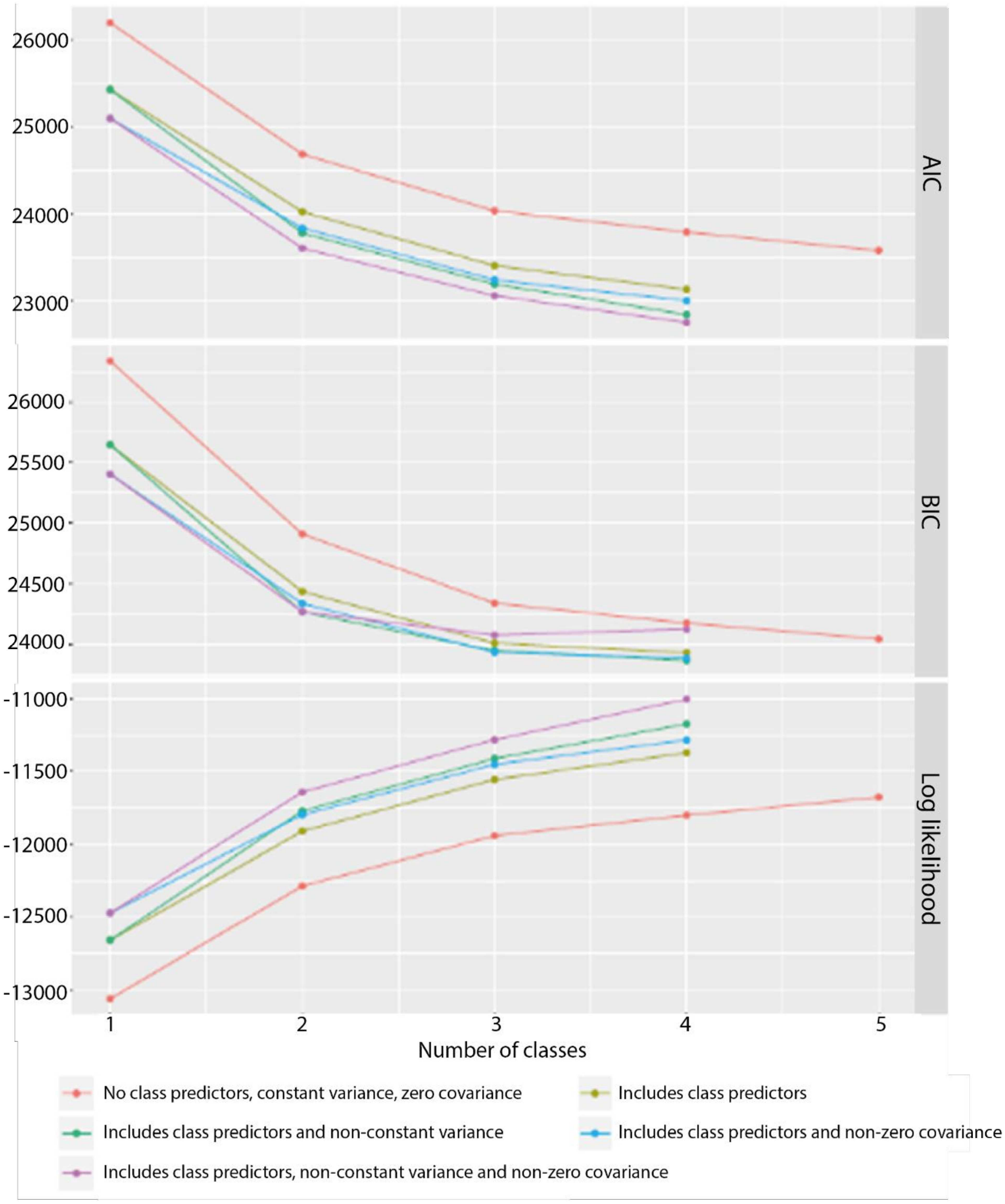

Figure S4: Plots of latent class analysis model fit indicators in the VANISH trial: a) Akaike Information Criterion (AIC); b) Bayesian Information Criterion (BIC) and c) log likelihood

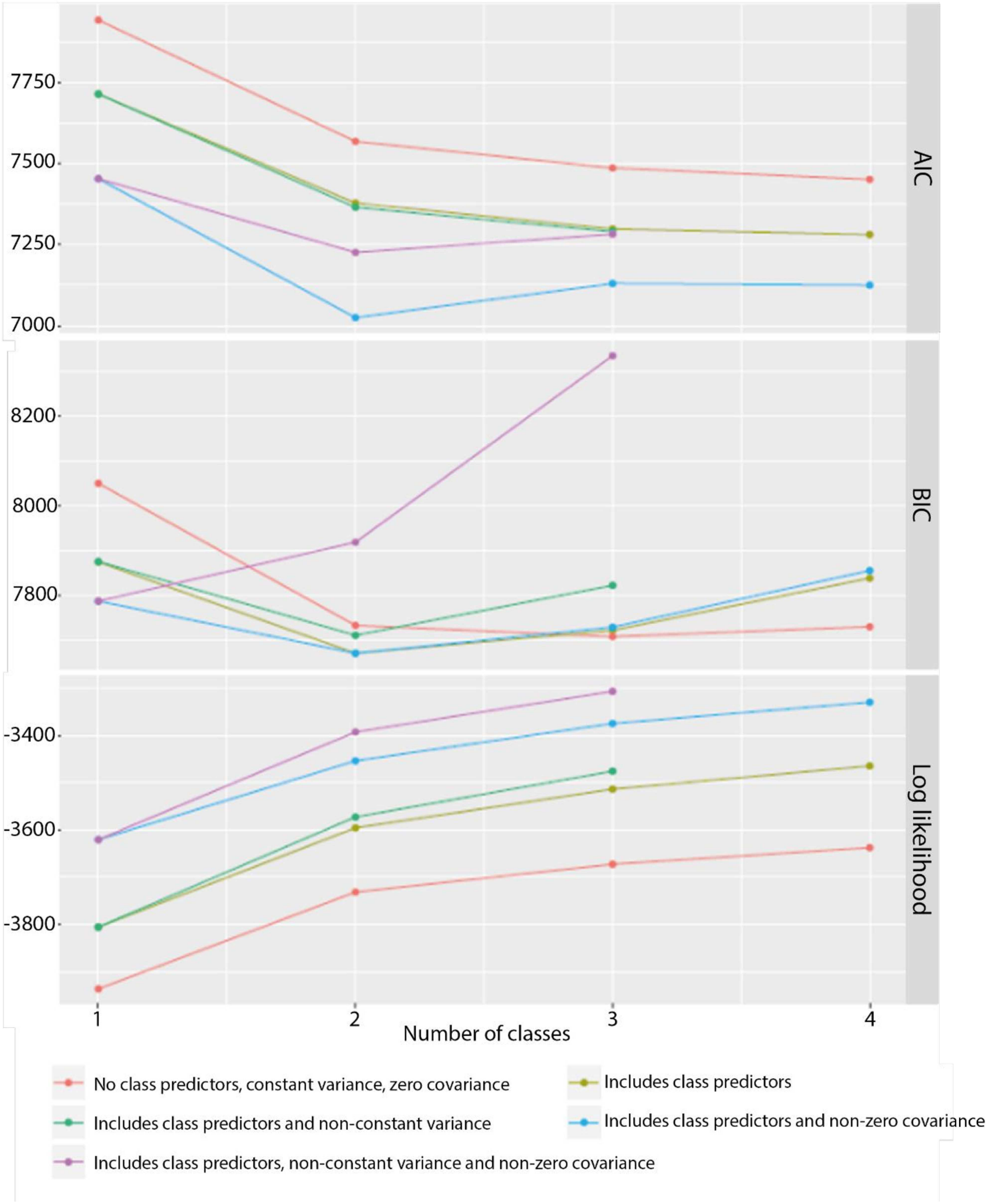

Figure S5 A) Forest plot showing treatment differences by latent class and trial for binary outcomes. LeoPARDS trial: 3-month survival, 28-day survival. VANISH trial: 28-day survival, 28-day survival free of renal failure. (V-N, vasopressin – noradrenaline; HC-P = hydrocortisone-placebo; RF, renal failure); B) Forest plot showing treatment differences by latent class and trial for continuous outcomes. LeoPARDS trial: mean daily SOFA score. VANISH: renal failure free days. (V-N, vasopressin – noradrenaline; HC-P = hydrocortisone-placebo). Treatment effects are expressed as a risk difference (RD), and the difference in treatment effects across classes as the difference in RD. 95% confidence intervals were calculated using linear regression with robust standard errors. Mean total SOFA is presented as the mean and standard deviation (SD), with differences between classes or treatment arms expressed as a difference in means. As mean total SOFA was skewed, 95% confidence intervals were calculated with bootstrapping. The median and interquartile range was presented for days alive and free of renal failure, again with bootstrap confidence intervals. For continuous variables, permutation tests were used to calculate p-values for the treatment-class interaction.

A

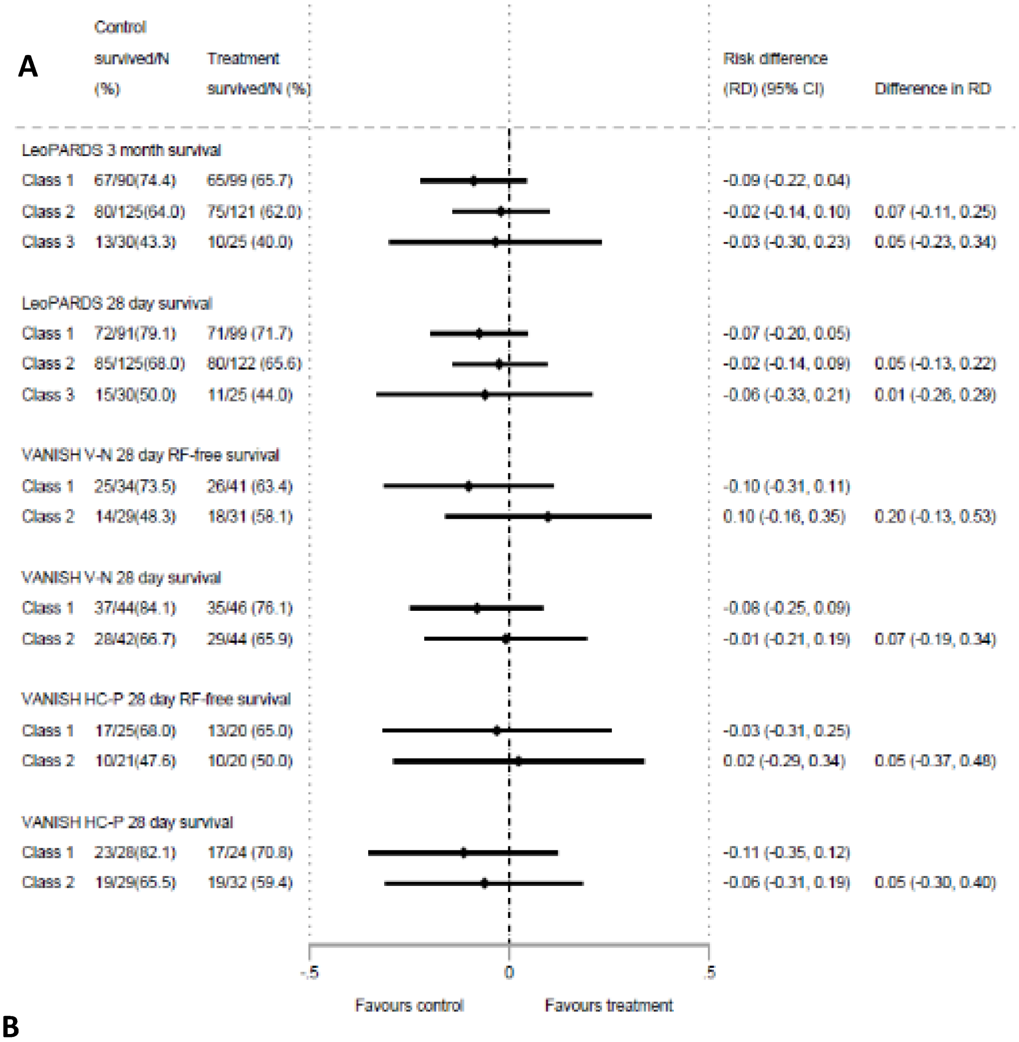
Control survived/N (%)
Treatment survived/N (%)
Risk difference (RD) (95% CI)
Difference in RD
LeoPARDS 3 month survival
Class 1 67/90(74.4) 65/99 (65.7) -0.09 (-0.22, 0.04)
Class 2 80/125(64.0) 75/121 (62.0) -0.02 (-0.14, 0.10) 0.07 (-0.11, 0.25)
Class 3 13/30(43.3) 10/25 (40.0) -0.03 (-0.30, 0.23) 0.05 (-0.23, 0.34)
LeoPARDS 28 day survival
Class 1 72/91(79.1) 71/99 (71.7) -0.07 (-0.20, 0.05)
Class 2 85/125(68.0) 80/122 (65.6) -0.02 (-0.14, 0.09) 0.05 (-0.13, 0.22)
Class 3 15/30(50.0) 11/25 (44.0) -0.06 (-0.33, 0.21) 0.01 (-0.26, 0.29)
VANISH V-N 28 day RF-free survival
Class 1 25/34(73.5) 26/41 (63.4) -0.10 (-0.31, 0.11)
Class 2 14/29(48.3) 18/31 (58.1) 0.10 (-0.16, 0.35) 0.20 (-0.13, 0.53)
VANISH V-N 28 day survival
Class 1 37/44(84.1) 35/46 (76.1) -0.08 (-0.25, 0.09)
Class 2 28/42(66.7) 29/44 (65.9) -0.01 (-0.21, 0.19) 0.07 (-0.19, 0.34)
VANISH HC-P 28 day RF-free survival
Class 1 17/25(68.0) 13/20 (65.0) -0.03 (-0.31, 0.25)
Class 2 10/21(47.6) 10/20 (50.0) 0.02 (-0.29, 0.34) 0.05 (-0.37, 0.48)
VANISH HC-P 28 day survival
Class 1 23/28(82.1) 17/24 (70.8) -0.11 (-0.35, 0.12)
Class 2 19/29(65.5) 19/32 (59.4) -0.06 (-0.31, 0.19) 0.05 (-0.30, 0.40)
-.5
0
.5
Favours control
Favours treatment

B

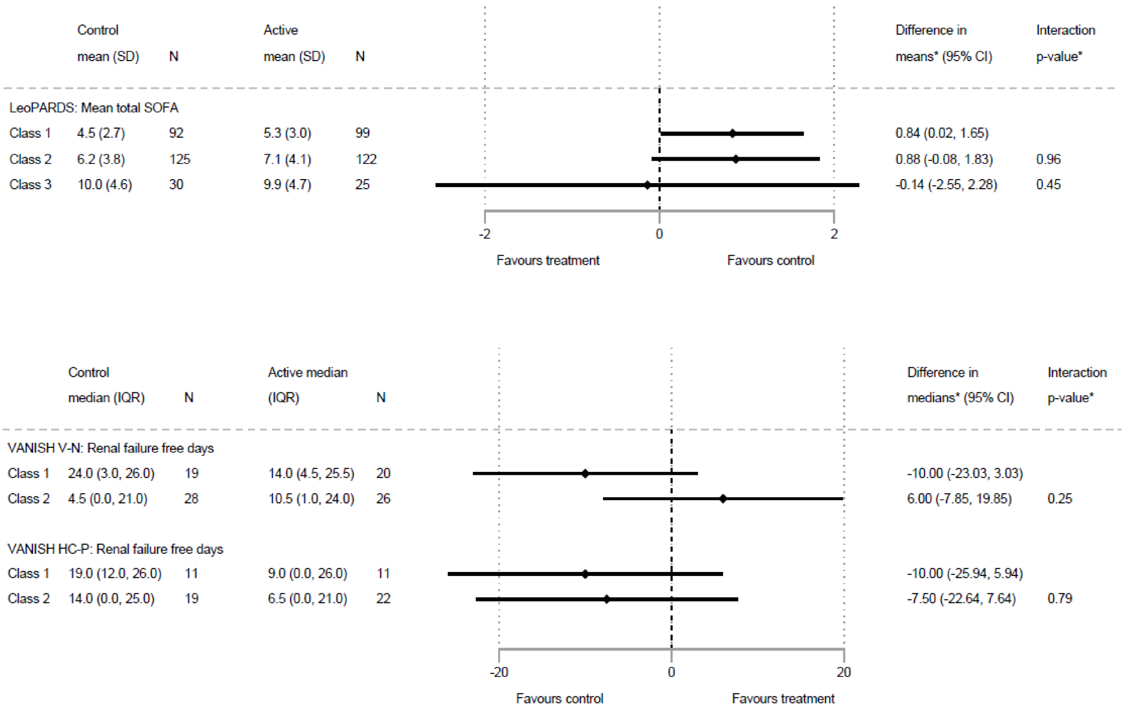
Control mean (SD) N
Active mean (SD) N
Difference in means* (95% CI)
Interaction p-value*
LeoPARDS: Mean total SOFA
Class 1 4.5 (2.7) 92 5.3 (3.0) 99 0.84 (0.02, 1.65)
Class 2 6.2 (3.8) 125 7.1 (4.1) 122 0.88 (-0.08, 1.83) 0.96
Class 3 10.0 (4.6) 30 9.9 (4.7) 25 -0.14 (-2.55, 2.28) 0.45
-2
0
2
Favours treatment
Favours control
Control median (IQR) N
Active median (IQR) N
Difference in medians* (95% CI)
Interaction p-value*
VANISH V-N: Renal failure free days
Class 1 24.0 (3.0, 26.0) 19 14.0 (4.5, 25.5) 20 -10.00 (-23.03, 3.03)
Class 2 4.5 (0.0, 21.0) 28 10.5 (1.0, 24.0) 26 6.00 (-7.85, 19.85) 0.25
VANISH HC-P: Renal failure free days
Class 1 19.0 (12.0, 26.0) 11 9.0 (0.0, 26.0) 11 -10.00 (-25.94, 5.94)
Class 2 14.0 (0.0, 25.0) 19 6.5 (0.0, 21.0) 22 -7.50 (-22.64, 7.64) 0.79
-20
0
20
Favours control
Favours treatment

Figure S6. Comparison of 28-day mortality of patients in GAinS and VANISH stratified by SRS, hierarchical cluster analysis groups and a combination of the sub-phenotypes. In VANISH the analysis has been performed in all patients and in only those who were randomized to and received placebo as the second study drugs due to our previous findings of an interaction between SRS sub-phenotypes and use of hydrocortisone on 28-day mortality that could influence mortality when combining sub-phenotypes (20) (SRS1, sepsis response signature 1; SRS2, sepsis response signature 2; LC, low cytokine cluster, HC, high cytokine cluster; LC-SRS2 patients in both the low cytokine cluster and the SRS2 sub-phenotype). Red bars - GAinS, pale blue bars - VANISH all patients, dark blue bars – the placebo arm from VANISH.

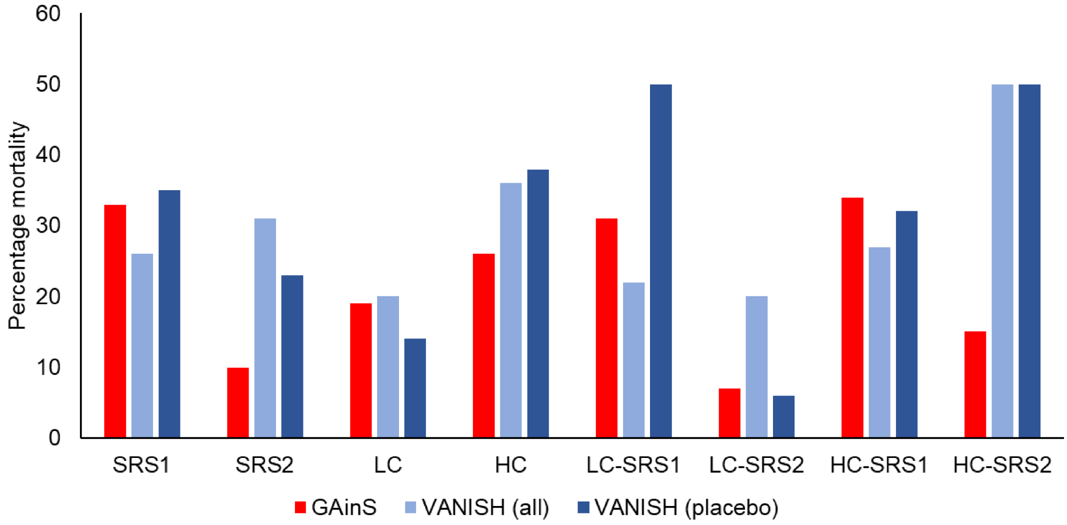

References

1. Bone, *et al.* Definitions for sepsis and organ failure and guidelines for the use of innovative therapies in sepsis. *Chest* 1992;101:1644–1655. doi: 10.1378/chest.101.6.1644.
2. Members of the American College of Chest Physicians/Society of Critical Care Medicine Consensus Conference. American College of Chest Physicians/Society of Critical Care Medicine Consensus Conference: definitions for sepsis and organ failure and guidelines for the use of innovative therapies in sepsis. *Critical care medicine* 1992;20:864–874. doi: 10.1097/00003246-199206000-00025.
3. Rautanen, *et al.* Genome-wide association study of survival from sepsis due to pneumonia: An observational cohort study. *The Lancet Respiratory Medicine* 2015;3:53–60. doi: 10.1016/S2213-2600(14)70290-5.
4. Davenport, *et al.* Genomic landscape of the individual host response and outcomes in sepsis: A prospective cohort study. *The Lancet Respiratory Medicine* 2016;4:259–271. doi: 10.1016/S2213-2600(16)00046-1.
5. Angus, *et al.* Severe community-acquired pneumonia: Use of intensive care services and evaluation of American and British Thoracic Society diagnostic criteria. *American Journal of Respiratory and Critical Care Medicine* 2002;166:717–723. doi: 10.1164/rccm.2102084.
6. Walden, *et al.* Patients with community acquired pneumonia admitted to European intensive care units: An epidemiological survey of the GenOSept cohort. *Critical Care* 2014;18:R58. doi: 10.1186/cc13812.
7. Tridente, *et al.* Patients with faecal peritonitis admitted to European intensive care units: An epidemiological survey of the GenOSept cohort. *Intensive Care Medicine* 2014;40:202–210. doi: 10.1007/s00134-013-3158-7.
8. Monti, *et al.* Consensus Clustering: A Resampling-Based Method for Class Discovery and Visualization of Gene Expression Microarray Data. *Machine Learning* 2003;52:91–118. doi: 10.1023/A:1023949509487.
9. Wilkerson, *et al.* ConsensusClusterPlus: a class discovery tool with confidence assessments and item tracking. *BIOINFORMATICS APPLICATIONS NOTE* 2010;26:1572–1573. doi: 10.1093/bioinformatics/btq170.
10. Charrad, *et al.* NbClust: An R Package for Determining the Relevant Number of Clusters in a Data Set. *Journal of Statistical Software* 2014;61:1–36. doi: 10.18637/jss.v061.i06.
11. R Core Team. *R: a language and environment for statistical computing*. R Foundation for Statistical Computing; 2015.
12. Shankar-Hari, *et al.* Defining phenotypes and treatment effect heterogeneity to inform acute respiratory distress syndrome and sepsis trials: secondary analyses of three RCTs. *Efficacy and Mechanism Evaluation* 2021;8:1–104. doi: 10.3310/EME08100.
13. Vermunt, *et al.* Latent class cluster analysis. *Applied Latent Class Analysis* 2002. p. 89–106.
14. Masyn. Latent Class Analysis and Finite Mixture Modeling. *The Oxford handbook of quantitative methods* 2013. p. 551.

15. Sinha, *et al.* Latent class analysis of ARDS subphenotypes: a secondary analysis of the statins for acutely injured lungs from sepsis (SAILS) study. *Intensive Care Medicine* 2018;44:1859–1869. doi: 10.1007/s00134-018-5378-3.

16. Calfee, *et al.* Acute respiratory distress syndrome subphenotypes and differential response to simvastatin: secondary analysis of a randomised controlled trial. *The Lancet Respiratory Medicine* 2018;6:691–698. doi: 10.1016/S2213-2600(18)30177-2.

17. Delucchi, *et al.* Stability of ARDS subphenotypes over time in two randomised controlled trials. *Thorax* 2018;73:439–445. doi: 10.1136/thoraxjnl-2017-211090.

18. Famous, *et al.* Acute respiratory distress syndrome subphenotypes respond differently to randomized fluid management strategy. *American Journal of Respiratory and Critical Care Medicine* 2017;195:331–338. doi: 10.1164/rccm.201603-0645OC.

19. Calfee, *et al.* Subphenotypes in acute respiratory distress syndrome: Latent class analysis of data from two randomised controlled trials. *The Lancet Respiratory Medicine* 2014;2:611–620. doi: 10.1016/S2213-2600(14)70097-9.

20. Antcliffe, *et al.* Transcriptomic signatures in sepsis and a differential response to steroids from the VANISH randomized trial. *American Journal of Respiratory and Critical Care Medicine* 2019;199:980–986. doi: 10.1164/rccm.201807-1419OC.

21. Burnham, *et al.* Shared and distinct aspects of the sepsis transcriptomic response to fecal peritonitis and pneumonia. *American Journal of Respiratory and Critical Care Medicine* 2017;196:328–339. doi: 10.1164/rccm.201608-1685OC.

22. Huber, *et al.* Variance stabilization applied to microarray data calibration and to the quantification of differential expression. *Bioinformatics* 2002;18:S96–S104. doi: 10.1093/bioinformatics/18.suppl_1.S96.

23. Johnson, *et al.* Adjusting batch effects in microarray expression data using empirical Bayes methods. *Biostatistics (Oxford, England)* 2007;8:118–27. doi: 10.1093/biostatistics/kxj037.

24. Westra, *et al.* MixupMapper: Correcting sample mix-ups in genome-wide datasets increases power to detect small genetic effects. *Bioinformatics* 2011;27:2104–2111. doi: 10.1093/bioinformatics/btr323.

25. Smyth. Linear models and empirical bayes methods for assessing differential expression in microarray experiments. *Statistical Applications in Genetics and Molecular Biology* 2004;3:Article3. doi: 10.2202/1544-6115.1027.

26. Fang, *et al.* XGR software for enhanced interpretation of genomic summary data, illustrated by application to immunological traits. *Genome Medicine* 2016;8:129. doi: 10.1186/s13073-016-0384-y.
